# IL-4 orchestrates STAT6-mediated DNA demethylation leading to dendritic cell differentiation

**DOI:** 10.1186/s13059-015-0863-2

**Published:** 2016-01-13

**Authors:** Roser Vento-Tormo, Carlos Company, Javier Rodríguez-Ubreva, Lorenzo de la Rica, José M. Urquiza, Biola M. Javierre, Radhakrishnan Sabarinathan, Ana Luque, Manel Esteller, Josep M. Aran, Damiana Álvarez-Errico, Esteban Ballestar

**Affiliations:** Chromatin and Disease Group, Cancer Epigenetics and Biology Programme (PEBC), Bellvitge Biomedical Research Institute (IDIBELL), 08908 L’Hospitalet de Llobregat, Barcelona, Spain; Present address: Bioinformatics Core, Centre for Genomic Regulation (CRG), 08003 Barcelona, Spain; Present address: Barts and The London School of Medicine and Dentistry, Centre for Neuroscience & Trauma, Blizard Institute, 4 Newark Street, London, E1 2AT UK; Present address: Nuclear Dynamics Programme, The Babraham Institute, Cambridge, CB22 3AT UK; Department of Experimental and Health Sciences, Barcelona Biomedical Research Park, Universitat Pompeu Fabra (UPF), 08003 Barcelona, Spain; Human Molecular Genetics Group, Bellvitge Biomedical Research Institute (IDIBELL), 08908 L’Hospitalet de Llobregat, Barcelona, Spain; Cancer Epigenetics Group, Cancer Epigenetics and Biology Programme (PEBC), Bellvitge Biomedical Research Institute (IDIBELL), 08908 L’Hospitalet de Llobregat, Barcelona, Spain

**Keywords:** Dendritic cell, differentiation, DNA demethylation, IL-4, STAT6, TET2

## Abstract

**Background:**

The role of cytokines in establishing specific transcriptional programmes in innate immune cells has long been recognized. However, little is known about how these extracellular factors instruct innate immune cell epigenomes to engage specific differentiation states. Human monocytes differentiate under inflammatory conditions into effector cells with non-redundant functions, such as dendritic cells and macrophages. In this context, interleukin 4 (IL-4) and granulocyte macrophage colony-stimulating factor (GM-CSF) drive dendritic cell differentiation, whereas GM-CSF alone leads to macrophage differentiation.

**Results:**

Here, we investigate the role of IL-4 in directing functionally relevant dendritic-cell-specific DNA methylation changes. A comparison of DNA methylome dynamics during differentiation from human monocytes to dendritic cells and macrophages identified gene sets undergoing dendritic-cell-specific or macrophage-specific demethylation. Demethylation is TET2-dependent and is essential for acquiring proper dendritic cell and macrophage identity. Most importantly, activation of the JAK3-STAT6 pathway, downstream of IL-4, is required for the acquisition of the dendritic-cell-specific demethylation and expression signature, following STAT6 binding. A constitutively activated form of STAT6 is able to bypass IL-4 upstream signalling and instruct dendritic-cell-specific functional DNA methylation changes.

**Conclusions:**

Our study is the first description of a cytokine-mediated sequence of events leading to direct gene-specific demethylation in innate immune cell differentiation.

**Electronic supplementary material:**

The online version of this article (doi:10.1186/s13059-015-0863-2) contains supplementary material, which is available to authorized users.

## Background

DNA methylation plays a fundamental role in differentiation by driving and stabilizing gene activity states during cell-fate decisions. DNA methylation maps at different steps of haematopoietic differentiation have yielded essential information about the different regulatory roles of DNA methylation in the various genomic regions (promoters, enhancers, etc.) that contribute to cell identity [1, 2] and support the notion that DNA methylation changes are tightly coupled to transcription factors (TFs) [3, 4]. However, we know little about the mechanisms directing the targeted deposition or erasure of DNA methylation, and the upstream mechanisms associated with them. Terminal differentiation from monocytes (MOs) to dendritic cells (DCs), macrophages (MACs) and other related cell types, like osteoclasts (OCs), represent ideal biological processes to investigate the mechanisms by which extracellular stimulation is translated in nuclear epigenetic control. Mononuclear phagocytes are crucial components of a wide range of important biological functions, such as the maintenance of homeostasis of several tissues, coordination of the innate immune response, and participation in adaptive immunity to proper activation, regulation and resolution [5]. These cells express high levels of the methylcytosine dioxygenase TET2 [6, 7], a key enzyme that successively oxidizes 5-methylcytosines, generating intermediate forms in the pathway towards demethylation [8], a process that takes place in these differentiation processes [6, 9]. In addition, the sets of TFs and upstream signalling pathways in DC and MAC differentiation have been well studied. This is useful for investigating the interplay between DNA methylation changes and TFs. By examining the differentiation of these closely related cell types, we can dissect the specific role and relationship between TFs and upstream signalling pathways and downstream interacting DNA methylation-related enzymes.

MOs, in response to inflammatory signals, such as those associated with bacterial infection [10], extravasate and are directed to inflamed peripheral tissues, where they terminally differentiate into MACs and/or DCs. This process occurs locally and is driven and determined by microenvironmental stimuli [11]. Although closely related, MO-derived DCs and MACs exert a variety of non-redundant functions as a result of activation of specific cell-restricted transcriptional programmes. MO conversion into inflammatory DCs or MACs can be recapitulated in vitro, by exposing cells to granulocyte macrophage colony-stimulating factor (GM-CSF)/interleukin 4 (IL-4) or GM-CSF only, respectively [12]. Human inflammatory DCs have recently been identified as the in vivo counterpart of in vitro GM-CSF/IL-4 MO-derived DCs [13]. The GM-CSF receptor activates JAK2 and downstream mediators in an inflammatory setting because GM-CSF constitutes a bona fide danger signal, advising that levels have exceeded steady state levels in conditions of inflammation or infection [14]. The IL-4 receptor (IL-4R) signals the activation of the Janus kinase 3 (JAK3)-STAT6 pathway through its common γ chain [15], which leads to the development of immature DCs. Full functionality is achieved in DCs and MACs upon maturation by engaging surface receptors, including several pattern recognition receptors such as Toll-like receptors (TLRs). Acting through outside-to-inside mechanisms, TLR-mediated signals shape a specific response aimed at triggering appropriate effector mechanisms to eliminate pathogens [16]. Bacteria-derived lipopolysaccharide (LPS) is a well-known maturation molecule that acts through TLR4 in MO-derived DCs and MACs in vitro and in vivo. Despite the recognition of the role of these and other factors in determining differentiation of these cell types, our knowledge of their contribution to the acquisition of functionally relevant epigenetic changes remains limited.

In the present work, we compared the specific DNA methylation changes associated with the differentiation from MOs to DCs and from MOs to MACs, as well as those occurring during the LPS-mediated maturation of these two cell types. These two differentiation processes differ only by the exposure of the former to IL-4, and so enabled us to determine the contribution of this external signal to DNA methylation changes downstream. Most changes occurred in the direction of DNA demethylation, during the differentiation step, whereas very few occurred during the LPS-mediated activation step. In contrast, thousands of genes became upregulated or downregulated in both the differentiation and maturation stages. Some of the DNA methylation and expression changes were common to DC and MAC differentiation, whereas others were specific to each differentiation process. In both cases, downregulation of TET2 impaired the acquisition of DNA demethylation and DC/MAC-specific surface markers, highlighting the functionality of DNA methylation changes. For a subset of genes, DNA demethylation during the differentiation step preceded any change in gene expression, which only occurred at the activation step, suggesting that DNA methylation changes prepare an epigenetic context suitable for the quick response necessary during activation. Most importantly, manipulation of the JAK3-STAT6 pathway downstream of IL-4 in DC differentiation altered the demethylation, expression and STAT6 binding in genes that are specifically demethylated in DCs. Inhibition of this pathway allowed demethylation of genes that are exclusively demethylated in MACs. Consistently, overexpression of a constitutively active form of STAT6 in MOs in the absence of IL-4 prevents specific demethylation of MAC genes at the time that promotes demethylation of DC-specific genes. This reveals the involvement of the IL-4-JAK3-STAT6 pathway in determining demethylation of DC-specific genes and preventing demethylation in MAC-specific genes. The results of our study enable us to identify for the first time the elements downstream of an external signal, in this case IL-4 in myeloid immune cells, that are translated into cell-type-specific functional DNA methylation changes essential for conferring identity and function.

## Results

### Differentiation from MOs to DCs and from MOs to MACs results in cell-type-specific demethylation of thousands of genes

**Fig. 1:**
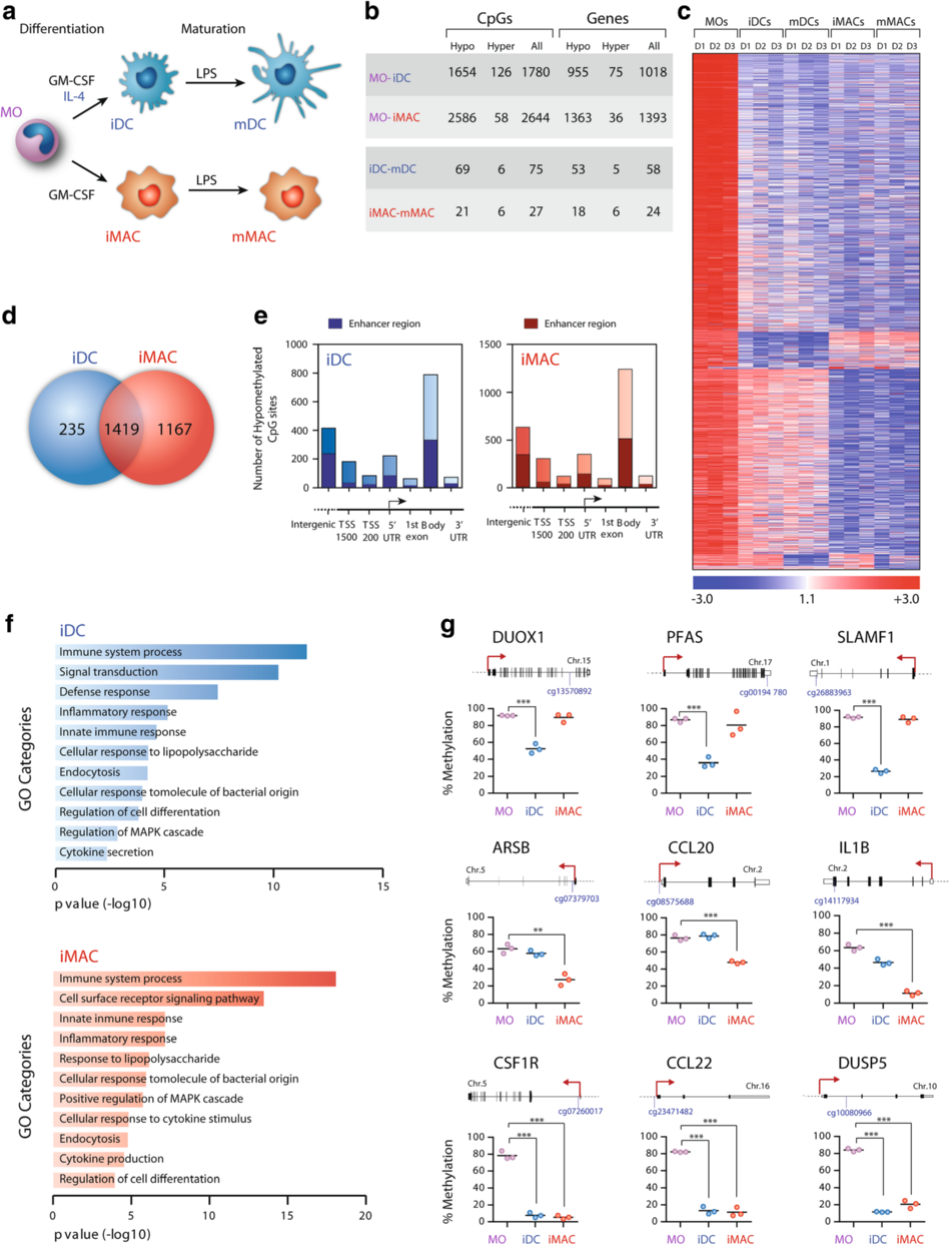
High-throughput DNA methylation comparison between monocytes (*MOs*) and derived dendritic cells (*DCs*) and macrophages (*MACs*). **a** Scheme depicting the differentiation system. Peripheral blood MOs were either exposed to granulocyte macrophage colony-stimulating factor (*GM-CSF*) + interleukin 4 (*IL-4*) or GM-CSF only to generate immature DCs and MACs (*iDCs* and *iMACs*), respectively. Maturation of these two cell types to mDCs and mMACs was achieved following incubation with lipopolysaccharide (*LPS*). **b** Summary of the DNA methylation changes obtained when comparing MOs differentiating to iDCs and iMACs, and the maturation towards mDCs and mMACs. Number of CpG sites and genes displaying significant gain (hyper) or loss (hypo) of DNA methylation changes are shown. **c** Heatmap of three paired samples (D1, D2 and D3) of MOs and their derived iMACs, iDCs, mMACs and mDCs. The heatmap includes all CpG-containing probes displaying significant methylation changes (>2-fold or <0.5-fold change; *p* < 0.01 and false discovery rate < 0.05) (data in Additional file 2). A scale is shown at the bottom, wherein beta values (i.e. the ratio of the methylated probe intensity to the overall intensity) range from −3 (lower DNA methylation levels, *blue*) to +3 (higher methylation levels, *red*). **d** Venn diagram showing the degree of overlap of demethylated CpGs/genes between MO-to-iDC and MO-to-iMAC differentiation. **e** Distribution of demethylated CpGs among genomic regions [intergenic, promoter (1,500 and 200 upstream of the transcription start site (*TSS*), 5′ untranslated region (*UTR*), first exon, gene body, and 3′UTR)] for MO-to-iDC and MO-to-iMAC differentiation. The darker insets within each bar indicate the number of sites annotated as enhancers. **f** Gene ontology (*GO*) enrichment analysis of demethylated CpGs in differentiation to iMACs and iDCs showing the most important categories. **g** Technical validation of the array data by bisulfite pyrosequencing of modified DNA. Three groups of genes are represented: demethylated genes specific to iDC differentiation, demethylated genes specific to iMAC differentiation, and genes that are commonly demethylated in iDC and iMAC differentiation

To dissect the downstream contribution of IL-4 in the acquisition of cell-type-specific DNA methylation changes in MO-to-DC differentiation, we generated three sets of matching samples corresponding to MOs from human peripheral blood, immature DCs (iDCs) and immature MACs (iMACs), following incubation of MOs with GM-CSF/IL-4 and GM-CSF only, respectively. Mature DCs (mDCs) and MACs (mMACs) were then created by exposing iDCs and iMACs to LPS (Fig. 1a). The comparison of MO-to-DC and MO-to-MAC differentiation allowed us to isolate the specific effect of IL-4, which is the differential factor in these two processes. We monitored these processes by testing different markers using RT-PCR (Additional file 1A) and fluorescence-activated cell sorting (FACS; Additional file 1B). For instance, quantitative RT-PCR demonstrated upregulation of DC markers (CD209) and mature DC markers (CD83), and that the level of expression of CD14 receptor was high in MOs, intermediate in MACs and low/negative in DCs. FACS analyses revealed that MOs were efficiently differentiated to iDCs (87–93 %, according to CD209 and CD206) and iMACs (88 %, according to CD206), and indicated a shift in the CD83 and CD86 markers that support efficient maturation of these cells, generating 79 % mDCs and 81 % mMACs (Additional file 1B).

We then performed DNA methylation profiling using bead arrays that interrogated the DNA methylation status of >450,000 CpG sites across the entire genome, covering 99 % of RefSeq genes. Statistical analysis of the combined data from the three biological replicates of MO-to-DC and MO-to-MAC revealed large changes in DNA methylation during the differentiation step (1,780 and 2,644 CpG sites, respectively). In contrast, only a few genes displayed differential DNA methylation during the maturation step (75 and 27 CpG sites for DC and MAC maturation, respectively) (Fig. 1b and Additional file 2). In all cases, demethylation prevailed over gains in DNA methylation, consistent with the results reported by others [6, 17, 18]. Specifically, demethylated CpG sites represented 92.9 % of total differentially methylated CpGs in MO-to-iDC (1,654 CpGs) and 97.8 % of differentially methylated CpG in MO-to-iMAC (2,586 CpGs). This contrasts with the findings in MO-to-OC differentiation, in which de novo deposition of the DNA methylation occurs to a similar extent as demethylation [9]. Changes corresponding to the average of three sample sets were almost identical to the pattern obtained for each individual sample, highlighting the specificity of the differences observed (Fig. 1c and Additional file 3A). The results for DC differentiation were similar to those reported by Zhang and colleagues [17] (78 % of the demethylated genes in their study were present in our own data, Additional file 3B).

Although most CpGs displaying a loss of methylation were common to the two differentiation processes (Fig. 1d), a significant fraction of demethylated CpGs were specific to each process: 14.2 % in MO-to-iDC differentiation (235 genes) and 45.1 % in MO-to-iMAC differentiation (1,167 genes). This implies that DNA demethylation may be important in determining the differences between the lineages. Given that IL-4 is the only cytokine to differ between these two processes, our findings suggest that events downstream of IL-4 may not only be responsible for the set of genes specifically demethylated in DCs, but also may directly block demethylation of those that are specific to MACs.

An analysis of the distribution of CpGs with a significant decrease in DNA methylation (Fig. 1e) revealed that most of them map to gene bodies (789 CpGs in MO-to-iDCs and 1,242 in MO-to-iMACs). Over 22 % were located at intergenic regions in both differentiation processes. Only about 15 % of the changes occurred near the transcription start site (TSS) (Fig. 1e). This reinforces the notion that a high proportion of the changes occur in regulatory regions outside promoters, such as enhancers located in the body of genes. Indeed, using the Illumina annotation tool we determined that 41 % and 43 % of all demethylated CpG sites in DC and MAC differentiation, respectively, are located in enhancers. The proportion of enhancers was particularly high in gene bodies and intergenic regions, as expected (Fig. 1e).

Gene ontology (GO) analysis of hypomethylated CpG revealed significant enrichment [false discovery rate (FDR) < 0.05] of a variety of functional categories important in iDC and iMAC differentiation and function, including inflammatory and innate immune response (Fig. 1f). These data suggest that DNA demethylation is targeted to genomic regions that are activated during DC and MAC differentiation.

As expected, we identified changes in several genes involved in DC and MAC function among the group of demethylated genes in both iDCs and iMACs (Additional file 4). For example, *CSF1R*, which codes for the receptor of the cytokine CSF1 involved in MAC differentiation, and *CCL22*, a cytokine that is released by DCs and MACs, were dramatically demethylated (Additional file 4). iMACs displayed specific demethylation on *CCL20*, an inflammation chemokine, and *IL1B*, a cytokine involved in immune and inflammatory response. We observed very specific demethylation in DC differentiation at a CpG site in the gene bodies of *DUOX1*, an oxidase involved in the antimicrobial-mediated response, and the signalling receptor *SLAMF1* (Additional file 4).

We then confirmed the robustness of the DNA methylation data in MO-to-iDC and MO-to-iMAC differentiation by bisulfite genomic pyrosequencing of CpG sites. The selection included genes that were demethylated in both differentiation processes (*CSF1R*, *CCL22*, *DUSP5*), and some that were only demethylated in MAC (*IL1B*, *ARSB*, *CCL20*) or DC differentiation (*SLAMF1*, *DUOX1*, *PFAS*). In all cases, bisulfite pyrosequencing confirmed the results of the beadchip array (Fig. 1g and Additional file 3C) and the demethylation at the aforementioned genes. It is well established that terminal differentiation of MOs into DCs/MACs occurs in the absence of cell division, indicating the occurrence of active DNA demethylation mechanisms. To further confirm this, we measured the extent of DNA replication in our DC and MAC differentiation experiments by treating cells with BrdU pulses. Consistent with previous observations [18], we found no significant differences between the negative control and the BrdU pulses, implying that DNA methylation changes observed during this period were independent of DNA replication (Additional file 1C). The participation of active DNA demethylation events in this process is reinforced by the previous findings of our and other groups [6, 9]. In fact, we observed changes in 5hmC, which is an intermediate oxidized base, resulting from TET2 activity and leading to active demethylation (Additional file 3D).

To test the implication and functionality of the methylcytosine dioxygenase TET2 in demethylation during DC and MAC differentiation, we downregulated TET2 levels using siRNA transfection against various TET2 sites and compared it to transfection with a control siRNA before DC/MAC differentiation was induced. TET2 downregulation partially impaired demethylation of both common and DC/MAC-specific genes. The impairment was partial because of a technical aspect related to the inability to achieve the maximum downregulation of TET2 before the differentiation processes had already started. In addition to the reduced demethylation, TET2 downregulation also resulted in a decrease in surface CD209 and CD83 markers; together with an increase in CD14 (which is higher in MOs than in DCs and MACs) (Additional file 5), demonstrating the functionality of DNA demethylation during these two processes.

### Expression changes and their relationship with DNA demethylation in MAC and DC differentiation and maturation

**Fig. 2:**
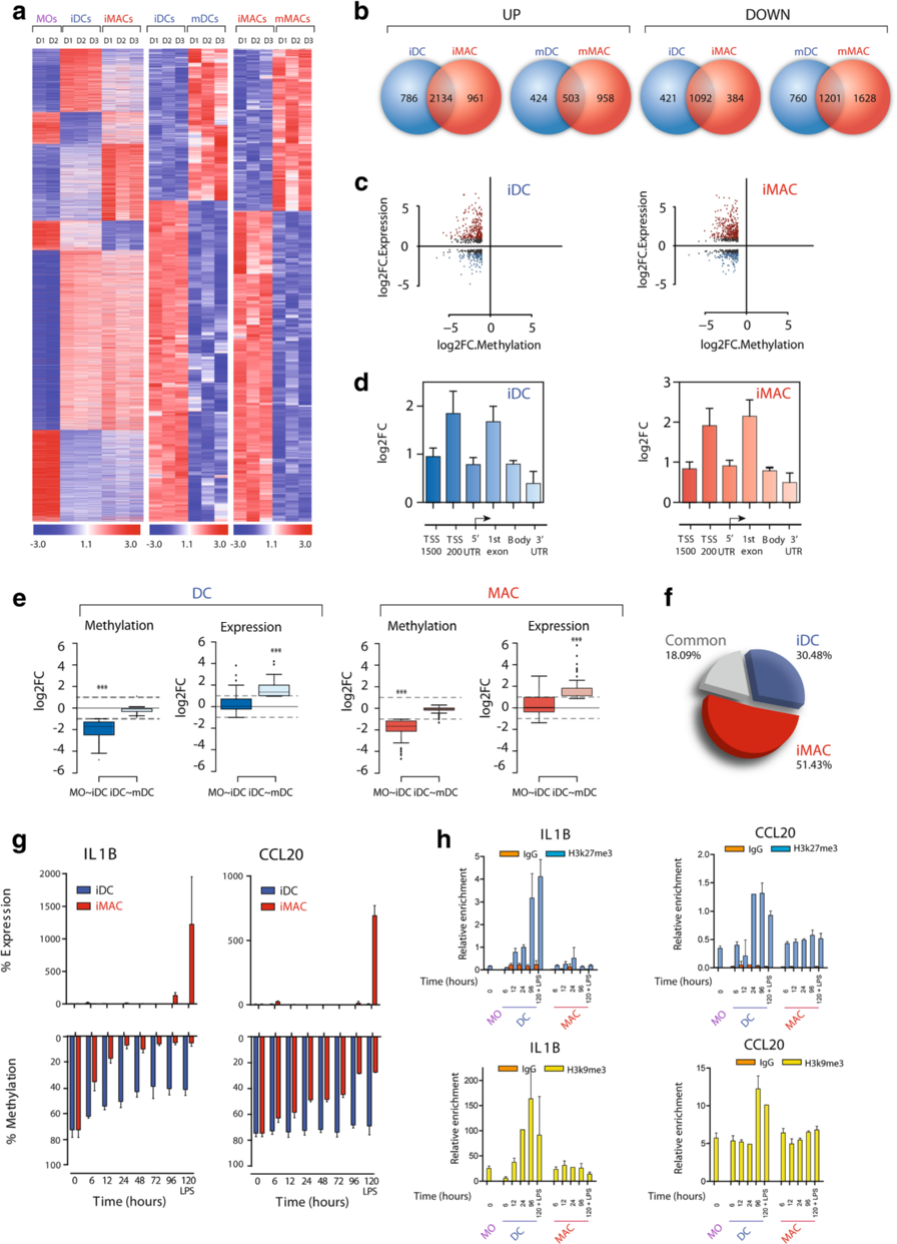
Comparison between methylation and expression data for macrophage (*MAC*) and dendritic cell (*DC*) differentiation and maturation. **a** Heatmap of significant changes between monocyte (*MOs*)-to-immature DC (*iDC*) and MO-to-immature MAC (*iMAC*) differentiation (*left*) and two additional heatmaps showing significant changes between iDC-to-mature DC (*mDC*) , and iMAC-to-mature MAC (*mMAC*) maturation. The heatmaps include all the genes displaying significant expression changes (>2-fold or <0.5-fold change; *p* < 0.01 and false discovery rate < 0.05). A scale is shown at the bottom, wherein expression values range from −3 (lower expression levels, *blue*) to +3 (higher expression levels, *red*). **b** Venn diagrams showing the degree of overlap of genes upregulated and downregulated between MO-to-iDC and MO-to-iMAC differentiation and iDC-to-mDC and iMAC-to-mMAC activation. **c** Scatterplots showing the relationship between the log_2_-transformed fold change (FC) in expression and the log_2_-transformed FC in DNA methylation. **d** Correlation between DNA methylation and expression data (the mean value for all CpG sites within a given sequence region is shown) for all the significantly demethylated genes organized by genomic location [intergenic, promoter (1,500 and 200 upstream of the transcription start site (*TSS*), 5′ untranslated region (*UTR*), first exon, gene body, and 3′UTR)]. **e** Box-plots representing the DNA methylation and expression values of all genes that are demethylated during DC and MAC differentiation and whose upregulation is stronger in the maturation step than in the previous differentiation step. **f** Diagram showing the proportion of genes among the DC-specific, MAC-specific and those that are demethylated in both processes with no significant changes in expression during the differentiation step and upregulated during the lipopolysaccharide (*LPS*)-dependent activation step. **g** Time-course analysis of DNA methylation and expression in two selected genes (*IL1B* and *CCL20*) during DC and MAC differentiation/maturation, among those displaying no changes in the differentiation step and a sharp increase in the maturation step. **h** Chromatin immunoprecipitation assays in *IL1B* and *CCL20* with anti-histone H3K27me3 and anti-histone H3K9me3 in MOs, and in a time-course manner in differentiation to iDCs and iMACs, as well as mDCs and mMACs (120 h + LPS). The Y-axis shows the relative enrichment in arbitrary units

To further investigate the functionality of DNA methylation changes, we generated expression profiles for the same cell types (MOs and derived iDCs, iMACs, mDCs and mMACs). We noted large changes in expression in both processes. Specifically, we observed upregulation of 2,920 and 3,095 genes and downregulation of 1,513 and 1,476 genes during the differentiation of MOs to iDCs and to iMACs, respectively (>2-fold change or <0.5-fold change; *p*-value < 0.01; FDR < 0.05) (Fig. 2a). We also identified large changes in the maturation process, whereby 927 and 1,461 genes were upregulated, and 1,961 and 2,829 were downregulated in the maturation from iDCs and iMACs to mDCs and mMACs, respectively, after LPS-mediated activation (Fig. 2a). Unlike changes in DNA methylation, which occurred primarily in the direction of demethylation and were concentrated in the differentiation of MOs to iDCs and iMACs, expression changes occurred in the direction of upregulation and downregulation, and large changes were observed during differentiation and maturation. A high proportion of expression changes were common to the processes of differentiation into DCs and MACs (Fig. 2b). Specifically, 73.12 % and 68.98 % of the upregulated genes and 72.24 % and 74.05 % of the downregulated genes were common to MO-to-iDC and MO-to-iMAC differentiation, respectively, whereas 54.37 % and 34.49 % of the upregulated genes and 61.09 % and 42.88 % of the downregulated genes were common to the two maturation processes.

To investigate the relationship between DNA methylation and expression changes, we compared the two data sets, focusing on genes that underwent significant demethylation. We found that DNA demethylation events were associated with both gene upregulation and downregulation (Fig. 2c), although most genes that became demethylated were overexpressed (70.4 % for MO-to-iDC and 67.1 % for MO-to-iMAC). We also examined whether the location of a given CpG site was related to the effects on expression. CpGs located in the TSS200 and the first exon had the strongest association between demethylation and overexpression (Fig. 2d) for both MO-to-iDC and MO-to-iMAC. Analysing the list of genes that were both demethylated and overexpressed during the differentiation step revealed the enrichment of categories of genes that are functionally relevant to DC and MAC biology (Additional file 6). For instance, we observed that genes in the inflammasome pathway that leads to IL1-mediated inflammation (including *PYCARD*, *IL1B* and *IL1A*, which act together during the MAC innate response [19]) were demethylated and overexpressed during MAC differentiation. The inflammasome sensor protein gene *AIM2* [20] was also demethylated during MAC differentiation and overexpressed in the MAC maturation step, strongly suggesting the need for additive signals to trigger this supramolecular inflammatory system.

As mentioned above, most DNA methylation changes occur at the differentiation level, both for DC and MAC differentiation, whereas large expression changes occur at the activation step, suggesting that a proportion of genes may undergo DNA methylation changes before their expression levels change. Indeed, we identified a set of genes for DC and MAC differentiation/maturation that became demethylated during differentiation but were only overexpressed at the maturation level (Fig. 2e), as if demethylation were priming these genes for upregulation for when they need to be expressed, that is, for when DCs or MACs encounter a compound such as LPS. Some of these genes were common to DCs and MACs, but others were specific to each cell type (Fig. 2f). Among these genes we identified some like *IL1B* and *CCL20* that undergo DNA demethylation during MAC differentiation, but only achieve overexpression in MACs following LPS treatment (Fig. 2g) (Additional file 6). In such cases, time-course analysis of histone modifications like H3K27me3 and H3K9me3 revealed that changes in these marks also precede LPS-mediated stimulation (Fig. 2h and Additional file 7), suggesting that other regulatory elements are directly responsible for activation of these genes once the chromatin context is suitable. Interestingly, the increase in these two heterochromatic marks took place in DCs, and not in MACs, where expression does not increase upon LPS-mediated stimulation.

Other genes had different relationships with DNA methylation changes, suggesting a variety of functional consequences associated with DNA demethylation observed at the differentiation step (Additional file 7).

### Inhibition of the JAK3-STAT6 pathway impairs DNA methylation and expression changes of DC-specific genes and is a positive switch for changes at MAC-specific genes

**Fig. 3:**
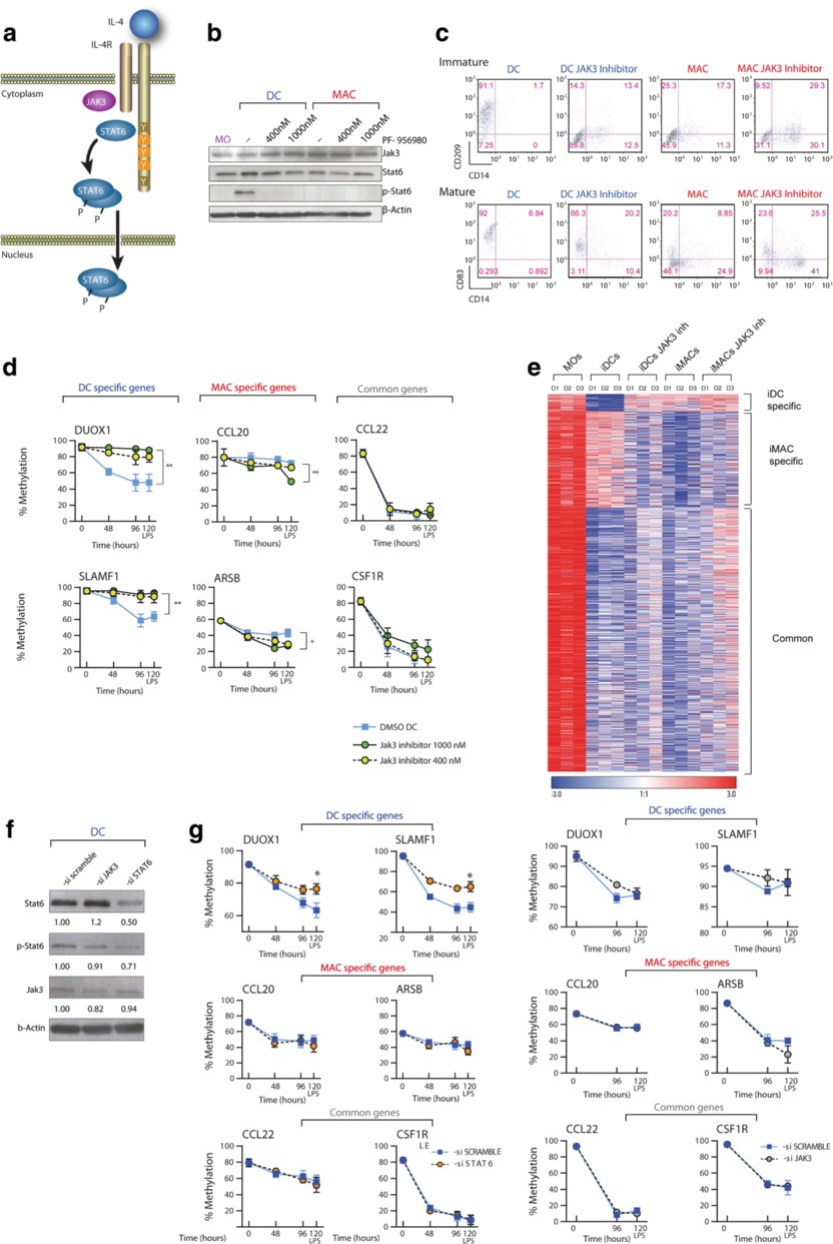
The IL-4-JAK3-STAT3 pathway has a direct role in targeting DNA demethylation in specific dendritic cell (*DC*) genes. **a** Diagram depicting the pathway downstream of IL-4, including IL-4 receptor (*IL-4R*), JAK3 and STAT6. IL-4 is able to activate and translocate STAT6 transcription factor to the nucleus, through STAT6 phosphorylation by JAK3 kinase. **b** Western blot assay confirming the presence of JAK3 and STAT6 in all cell types studied and the exclusive existence of the phosphorylated form of STAT6 in DCs. Inhibitor PF-956980 at a concentration of 400 and 1,000 nM throughout the entire differentiation process is able to prevent STAT6 phosphorylation. **c** Effects of PF-956980 on CD209 and CD14 in both granulocyte macrophage colony-stimulating factor (*GM-CSF*)/IL-4-stimulated and GM-CSF-stimulated cells at 96 h, as measured by fluorescence-activated cell sorting. **d** Effects of JAK3 inhibition by PF-956980 on DNA methylation over time in DC (GM-CSF/IL-4) differentiation, focusing on two DC-specific genes (*left*), macrophage (*MAC*)-specific genes (*centre*) and two genes demethylated in both DC and MAC differentiation (*right*). **e** Heatmap showing the effect in DNA methylation of JAK3 inhibition by PF-956980. This treatment is able to erase the DC-specific methylation signature in monocytes (*MOs*) treated with GM-CSF/IL-4. **f** Western blot assays showing levels of STAT6, phospho-STAT6 and JAK3, 96 h after treatment with GM-CSF/IL-4 and transfection with siRNA for STAT6. B-actin has been used as loading control. **g** Effects of siRNA against STAT6 (*left panel*) and JAK3 (*right panel*) on DNA methylation changes over time focusing on two DC-specific genes (*top*), MAC-specific genes (*middle*) and two genes demethylated in both DC and MAC differentiation (*bottom*). *DMSO* dimethyl sulfoxide, *iDC* immature dendritic cells, *iMAC* immature macrophage, *LPS* lipopolysaccharide

IL-4 signalling is crucial and indispensable to the development of human MO-derived DCs. One of the most important outcomes of our DNA methylation analysis was the identification of a subset of genes that are specifically demethylated in DC differentiation in response to IL-4. To address the role of IL-4 in driving these DC-specific DNA methylation changes, we studied the contribution of signalling mediators downstream of IL-4R. Membrane-bound type I IL-4R activates the tyrosine kinase JAK3, which phosphorylates STAT6 at Tyr641, leading to its translocation to the nucleus and binding to target genes [21–23] (Fig. 3a). To examine the role of the IL-4-JAK3-STAT6 pathway in the acquisition of DC-specific DNA methylation and expression changes, we first tested the impact of JAK3 inhibition on the regulation of the aforementioned genes. To this end, we first used a JAK3-selective inhibitor, PF-956980 [24]. We differentiated MOs to DCs and MACs with two different concentrations of PF-956980 to select the conditions under which it is active. STAT6 phosphorylation, which renders STAT6 into its active form, is only present under the conditions for DC differentiation and not for MAC differentiation (when IL-4 is absent). As expected, STAT6 phosphorylation disappeared following JAK3 inhibition with 400 nM and 1,000 nM PF-956980 (Fig. 3b). In the case of MACs, we did not observe STAT6 phosphorylation, given the lack of stimulation of JAK3, and therefore the addition of PF-956980 did not make any difference (Fig. 3b). Treatment with PF-956980 affected the presence of the surface markers CD209 and CD83 during GM-CSF/IL-4-mediated differentiation to DCs (Fig. 3c and Additional file 8), resulting in the generation of profiles closer to those displayed by MACs. This demonstrates the functional effects of PF-956980 in inhibiting DC differentiation.

The effect of PF-956980 was very specific to the impairment of demethylation of DC-specific genes in DC differentiation (Fig. 3d), and had little effect on the demethylation of MAC-specific genes in MAC differentiation or in genes that are commonly demethylated in both DC and MAC differentiation (Fig. 3d and Additional file 8B). Interestingly, in the presence of JAK3 inhibitors and under the conditions required for DC differentiation, DNA methylation levels of genes that were specifically demethylated in MO-to-iMAC differentiation resembled those observed in the absence of IL-4, indicating that inhibition of the pathway downstream of IL-4 removed the constraints on this set of genes towards their DNA demethylation under the standard conditions for DC differentiation (Fig. 3d).

In general, the effects at the expression level were as expected, and impaired DNA demethylation was associated with diminished overexpression of DC-specific genes during differentiation. Most notably, we observed impaired overexpression of genes that only underwent expression changes in the maturation step, once DNA demethylation had been inhibited through the action of JAK3 inhibitors (Additional file 8C).

To explore the extent of the role of the IL-4-JAK3-STAT6 pathway in the acquisition of the DC-specific methylation signature, we performed a new methylation profiling to test the effects of inhibiting JAK3. A comparison of MOs with MOs differentiated to iDCs and iMACs both in the presence and absence of the JAK3 inhibitor PF-956980 revealed that the DNA methylation patterns of iDCs incubated with PF-956980 cluster together with iMACs (Additional file 8D). In other words, treatment with PF-956980 erases the DC-specific signature, and renders a DNA demethylation pattern indistinguishable to that of iMACs (Fig. 3e). The specific analysis of some of the previously studied genes confirmed this effect (Additional file 8E).

To unequivocally test the potential causal relationship between JAK3 and STAT6 in the demethylation of DC-specific genes, we investigated the consequences of ablating JAK3 and STAT6 expression in MOs. We downregulated JAK3 and STAT6 levels in MOs using transient transfection experiments with siRNA cocktails that target different sites for each of these two proteins in comparison with a control siRNA. Twenty-four hours after transfection, we induced DC differentiation with GM-CSF/IL-4. Under these conditions, we used a western blot to check the effects on JAK3 and STAT6 levels 4 days after GM-CSF/IL-4 stimulation of MOs. This method enabled us to confirm that the STAT6 and JAK3 were downregulated by close to 50 % and 20 % (Fig. 3f), respectively. As a result, we observed a noticeable shift of the surface DC markers CD209 and CD83 (Additional file 9A).

We then checked the effects of JAK3 and STAT6 depletion on the demethylation of DC-specific, MAC-specific and DC/MAC common genes. Similar to the results obtained from the pharmacological inhibition of JAK3, siRNA-mediated depletion of JAK3 and STAT6 very specifically impaired the demethylation of DC-specific genes in DC differentiation (Fig. 3g) and had little effect on the demethylation of MAC-specific genes in MAC differentiation (Additional file 9B) or in genes that are commonly demethylated in both DC and MAC differentiation. These results not only confirmed the participation of JAK3, downstream to IL-4, in the demethylation of DC-specific genes, but also the participation of STAT6, the target of JAK3.

### Constitutively activated STAT6 induces demethylation of DC-specific genes during GM-CSF-only differentiation

**Fig. 4:**
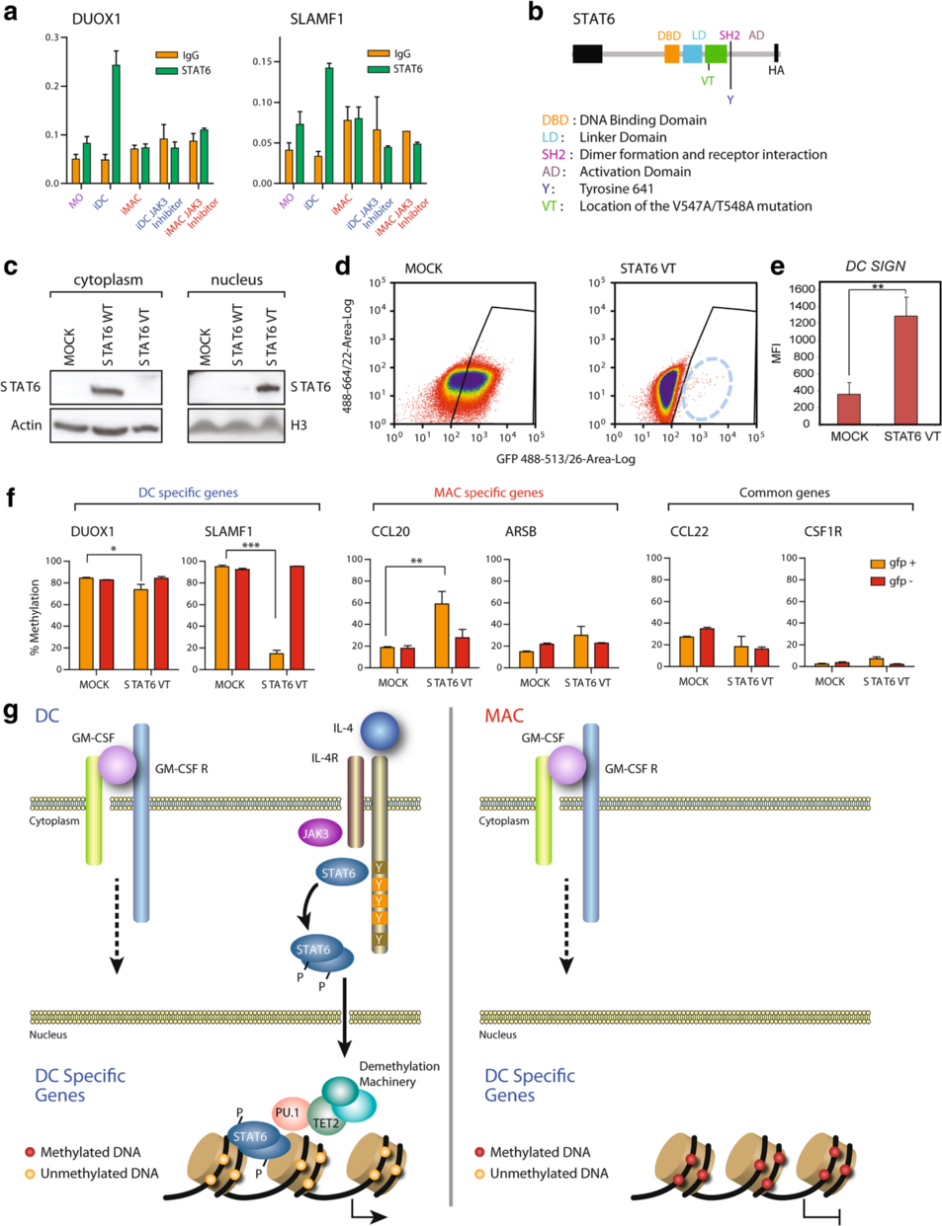
Direct involvement of STAT6 in targeting DNA demethylation in specific dendritic cell (*DC*) genes. **a** Chromatin immunoprecipitation assays confirm STAT6 binding to two genes (*DUOX1*, *SLAMF1*) that become specifically demethylated in DC differentiation. The panel also shows the loss of STAT6 to DC-specific genes following treatment with JAK3 inhibitor PF-956980. Lack of binding of STAT6 under the conditions of macrophage (*MAC*) differentiation (also in the presence of PF-956980) is also shown. **b** Diagram showing STAT6 gene domains and the double mutant that mimics phosphorylated STAT6 (*STAT6VT*) and leads to a gain of function. **c** Western blot of 293 T cells transfected with wild-type STAT6 (*STAT6 WT*) and the activating double mutant STAT6VT showing the presence of the protein in the cytosolic or nuclear fraction. **d** Fluorescence-activated cell sorting analysis showing the green fluorescent protein (*GFP*) labelling of cells infected with a GFP-expressing lentiviral MIG vector (pCDH-MIG) containing STAT6VT (*right panel*) and empty GFP-expressing MIG vector as a negative control (*left panel*). **e** CD209 mean fluorescence measured by flow cytometry in monocytes (*MO*) infected with STAT6 VT and the MOCK control vector after 9 days of culture with granulocyte macrophage colony-stimulating factor (*GM-CSF*). Analysis was done by setting a gate to select cells with high GFP expression. **f** Effects on the DNA methylation levels of two DC-specific genes in MOs treated with GM-CSF for 48 h followed by infection with mock or STAT STAT6V. Both the GFP+ and GFP− fractions are shown. Examples of two DC-specific genes (*left*), MAC-specific genes (*middle*) and two genes demethylated in both DC and MAC differentiation (*right*) are shown. **g** Model depicting the participation of the IL-4-JAK3-STAT6 pathway in targeting demethylation of DC-specific genes

To further investigate the potential direct involvement of STAT6 in the demethylation of DC-specific genes, we performed chromatin immunoprecipitation (ChIP) assays with STAT6. We found that STAT6 did interact specifically with DC-specific genes like *DUOX1* and *SLAMF1* in DC differentiation (Fig. 4a), whilst there was no binding of these genes during MAC differentiation. Interestingly, pharmacological inhibition of JAK3 led to impaired binding of STAT6 in DC-specific genes (Fig. 4a), reinforcing the notion of the dependence on IL-4 and JAK3 for this interaction. We then investigated whether STAT6 interacts with TET2, either directly or through other intermediates such as PU.1. However, we were unable to identify any direct interaction between STAT6 and TET2 (not shown). It should be noted that these experiments are technically challenging, and such an interaction cannot be fully discounted. An alternative mechanism may be provided if STAT6 recruits PU.1, which has in turn been proven to recruit TET2 [9] in a related MO differentiation process. In fact, synergism between STAT6 and PU.1 has been previously shown [25]. To test whether PU.1 participates in demethylating these genes, we also performed siRNA experiments against PU.1. We determined that PU.1 downregulation also impairs demethylation of some of these genes, although in a less specific manner than STAT6. In addition, we also observed an effect on the surface markers of both DCs and MACs (Additional file 10).

To conclusively establish the role of IL-4/JAK3-dependent demethylation of DC-specific genes via STAT6, we performed gain-of-function experiments in MOs stimulated exclusively with GM-CSF and transfected with a constitutively activated form of STAT6. STAT6VT carries two amino acid changes in the SH2 domain that affect the overall structure and stability of the monomeric and dimeric protein [26] (Fig. 4b). When overexpressed in mammalian cells, STAT6VT undergoes tyrosine phosphorylation; is translocated to the nucleus (Fig. 4c), where it binds DNA; and activates transcription in an IL-4-independent manner. We infected MOs with a green fluorescent protein (GFP)-expressing lentiviral MIG vector (pCDH-MIG) containing STAT6VT and, in parallel, an empty GFP-expressing MIG vector as a negative control. Following infection, we stimulated cells with GM-CSF in the absence of IL-4, that is, under our conditions for MAC differentiation. Infection of MOs with the GFP-expressing empty vector achieved higher levels than those with STAT6VT GFP vector, probably due to the lower titre of lentiviruses containing a larger construct (Fig. 4d). In any case, we were able to isolate GFP+ cells in both conditions, following 9 days after GM-CSF stimulation. Not surprisingly, the ectopic expression of STAT6VT resulted in increased levels of the DC-specific marker DC-sign, following GM-CSF stimulation and in the absence of IL-4 (Fig. 4e). We then performed bisulfite pyrosequencing of DC-specific and MAC-specific genes and found that STAT6VT overexpression was able to induce demethylation of DC-specific genes, such as *DUOX1* and *SLAMF1*, by-passing IL-4R upstream signalling (Fig. 4f). In addition, the MAC-specific genes *CCL20* and *ARSB*, which are normally demethylated in the presence of GM-CSF, did not become demethylated under the presence of STAT6VT (Fig. 4f), strongly indicating that STAT6 prevents their demethylation under the conditions of DC differentiation. In contrast, genes that were demethylated under our standard DC and MAC differentiation conditions (like *CCL22* and *CSF1R*) were not affected by the overexpression of STAT6VT. In summary, STAT6 is not only responsible for demethylating DC-specific genes but also for preventing demethylation of MAC-specific genes.

Altogether, our results demonstrate a direct relationship between the extracellular stimulation through IL-4 leading to MO-to-DC differentiation and the acquisition of DC-specific DNA methylation and expression patterns, together with the inhibition of MAC-specific genes. Moreover, we prove the role of the IL-4-JAK3-STAT6 pathway in instructing the cell epigenome to engage a specific differentiation state towards DCs, at the expense of MAC differentiation.

## Discussion

Our results identify for the first time the sequence of events that occur downstream of a cytokine when instructing specific TET2-mediated active DNA demethylation associated with the differentiation of effector cells of the innate immune response. Specifically, we have established that IL-4 targets a demethylation signature of a specific subset of genes in DC differentiation (and also prevents demethylation of inappropriate MAC-specific genes) in a STAT6-dependent manner, providing a direct causal relationship between external stimulation by this cytokine and the targeted epigenetic changes that are necessary for the acquisition of identity. These changes, as well as the majority of DNA methylation changes in DC and MAC differentiation, occur during the differentiation step from MOs, before LPS-induced maturation. The direction of DNA methylation changes in DC and MAC, as well as their occurrence during the differentiation step, contrast with the expression changes that occur in both steps and both directions (upregulation and downregulation). We have identified a set of genes in which DNA demethylation precedes their upregulation, indicating that DNA demethylation prepares those genes for subsequent overexpression during the maturation step.

In accordance with the findings of others [6], differentiation from MOs to DCs and from MOs to MACs is associated with a predominant occurrence of DNA demethylation. Conversely, very few DNA demethylation changes occur during the activation of these two cell types when exposed to LPS, which activates these cells to respond through TLR4 and CD14. The predominance of demethylation changes differs from the direction of changes in a related model, specifically the M-CSF/RANKL-mediated differentiation of MOs to OCs, in which gains of both DNA methylation and demethylation occur to a similar extent [9]. As in the cases of MO-to-DC and MO-to-MAC differentiation, demethylation in OC differentiation takes place through active mechanisms [6, 9] and occurs to a similar extent. In contrast, unlike in DC and MAC differentiation, where gain of DNA methylation is restricted to a few genes, thousands of CpG sites become hypermethylated in OC differentiation. It is likely that the widespread occurrence of deposition of DNA methylation in OC differentiation is due to the fact that OC differentiation takes place over a few weeks. It is also remarkable that the vast majority of DNA demethylation events in DC and MAC differentiation/maturation occur in the differentiation step and that only a few changes take place during the maturation of these cells following exposure to LPS. If DNA methylation changes have an effect on the stability of cell identity, it might simply reflect that this control is more important in the initial differentiating step, from MOs to iDCs/iMACs, as it also prepares those cells and their chromatin for a potentially rapid response to fulfil their function in immunity in case of an insult.

The gene expression data show that large changes in upregulation and downregulation occur in the differentiation and maturation steps, reflecting the activation of specific response transcriptional programmes. In a general way, demethylation is associated with transcriptional activation, whereas gain of methylation is often associated with gene silencing. Although the current view is now more complex and involves different types of relationship between DNA methylation and expression status, our data on MO-to-DC and MO-to-MAC differentiation also show an association between demethylation and gene activation that is particularly evident in demethylated CpGs near the TSS and the first exon of genes. Identifying genes displaying both demethylation and overexpression during differentiation revealed an enrichment of genes relevant to DC/MAC function. However, it is of particular note that there is a set of genes that undergo large changes in gene expression (particularly genes that become overexpressed) only during the maturation step, although their demethylation occurred previously, during differentiation. It is as if their demethylation were a prerequisite for their subsequent overexpression, and a second signal were needed ultimately to trigger the activation once the unmethylated/competent status has been achieved. This separation between demethylation and overexpression in the differentiation and maturation steps may constitute a mechanism to facilitate a rapid response following activating stimuli, such as an encounter with a bacterial antigen, while keeping a threshold that prevents improper triggering. In this respect, demethylation preceding stimuli-mediated activation may act both as a gate-keeper and a facilitator of stimulus-dependent gene expression. A preparation of the genome in non-cycling cells has recently been described in naïve B cells, whereby single-stranded DNA sequencing experiments showed that most of the genome is poised for antigen-driven activation [27].

Comparing two in vitro differentiation models that only differ in the participation of the cytokine IL-4 allowed us to directly assess IL-4’s involvement in the DNA methylation changes underlying the fine-tuning of cell fate acquisition. Differentiation to DCs and MACs involves demethylation of a large common set of genes, while other CpGs become demethylated in a DC-specific and MAC-specific manner. The acquisition of DC or MAC fate in our model differed only with respect to exposure to IL-4, so we reasoned that the difference is largely determined by events downstream of IL-4R. It is conceivable that IL-4-dependent cell specification of DC identity relies not only on determining which genes are specifically demethylated in DCs, but also on preventing demethylation of those that are specifically demethylated in MAC differentiation. It was reported some time ago that IL-4 inhibits the production of tumour necrosis factor alpha (TNF-ɑ), IL-1 and prostaglandin E2 in human MOs [28] in a STAT6-dependent and a STAT6-independent manner [29]. Our results could provide molecular evidence of such inhibition, given that IL-1a, IL-1b and TNF all become demethylated in our system.

Human MOs bind IL-4 to membrane-bound dimeric IL-4R, which consists of the IL-4Rɑ chain that recognizes IL-4 with high affinity [30], and a second chain that forms either Type I (with γc chain) or Type II receptors (with IL-13Rɑ1). Upon dimerization, signal transduction leads to the activation of several routes, with STAT6 activation and translocation to the nucleus following JAK3-mediated phosphorylation as a hallmark [31]. Our study has shown that the IL-4-JAK3-STAT6 pathway plays a major role in the specific methylation changes that drive DC differentiation (Fig. 4g). We have demonstrated that JAK3 and STAT6 downregulation impairs DC-specific demethylation and that the ectopic expression of a constitutively activated/nuclear form of STAT6 leads to specific demethylation of DC genes under the conditions of MAC differentiation (in the absence of IL-4). These results suggest a direct role of IL-4-JAK3-STAT6 in promoting specific demethylation and subsequent activation of a subset of DC genes, as well as impairing the demethylation of MAC-specific genes. The inhibitory effect of STAT6 has been described in Th2 differentiation of human T cells in which STAT6 regulates the expression of around 80 % of IL-4-responsive genes [32]. Our results are in line with a recently proposed model of asymmetric participation of different STATs in response to combinations of cytokines, which strongly suggests that in response to two cytokine signals, one STAT may provide a wider transcriptional programme that is restricted to gain specificity by the superimposed action of another STAT [33]. In the present work, we extend this notion to epigenetic regulation, in particular, DNA methylation.

Given the participation of TET2 in the active demethylation of DC and MAC differentiation, as shown in this study, it seemed likely that STAT6 would recruit this enzyme. However, immunoprecipitation experiments were unable to demonstrate such interaction. Because PU.1 has been shown to associate and recruit TET2 to genes that become demethylated [9], and also interacts with STAT6 [25], a possible scenario could involve STAT6 recruitment of PU.1-TET2 to genes that become specifically demethylated in DCs (Fig. 4g). It is likely that other TFs also participate in this process. In fact, there are various connections and mechanisms that associate DNA methylation changes with TF binding [34]. Our analysis of the enrichment of TF-binding motifs near demethylated CpGs supports this notion: some of them, like the GATA1 binding motif, are specifically enriched in genes demethylated in DCs, whereas C/EBPα/β, MITF, NANOG and CREB are enriched at the demethylated sites in iMAC differentiation. However, the participation of these factors in DNA methylation could also be indirect. It will be interesting to establish whether STAT6, or any other additional TF, is able to directly recruit TET proteins to the sites undergoing DNA demethylation.

The finding that a cytokine like IL-4 drives the DNA demethylation of specific sets of genes that are crucial for DC versus MAC identity and function opens up a number of possibilities from the fundamental and translational points of view, as new targets for pharmacological intervention of innate immune cell responses.

## Conclusions

In the present study, we have compared the DNA methylation changes during human MO-to-DC and MO-to-MAC differentiation, in which IL-4 represents the sole differential factor determining DC versus MAC fate. Our data reveal the existence of both common and cell-type specific DNA demethylation of many genes, and that such DNA demethylation depends on TET2 and is essential for the acquisition of proper DC and MAC identity. We demonstrate that upon IL-4R engagement by its ligand, activation of the JAK3-STAT6 pathway leads to the acquisition of DC-specific demethylation and expression profiles, by activating DC-specific genes and repressing MAC-specific genes. Furthermore, we show that IL-4R signalling can be bypassed with the introduction of a constitutively activated STAT6 form that instructs DC-specific methylation changes in the absence of IL-4. In summary, our results constitute the first report of a cytokine-mediated downstream sequence of events that leads to direct gene-specific demethylation in innate immune cell differentiation.

## Methods

### Differentiation of DCs and MACs from peripheral blood mononuclear cells

Human samples (blood) used in this study came from anonymous blood donors and were obtained as buffy coats from the Catalan Blood and Tissue Bank (Banc de Sang i Teixits) in Barcelona. The anonymous blood donors received oral and written information about the possibility that their blood would be used for research purposes, and any questions that arose were then answered. Before providing the first blood sample, all donors signed a consent form at the Banc de Sang. The Banc de Sang follows the principles set out in the WMA Declaration of Helsinki. The protocol used to isolate and differentiate cells from these anonymous donors was approved by the Ethics Committee of the University Hospital of Bellvitge (CEIC) on 28 May 2011 (and renovated on 4 December 2014).

The blood was carefully layered on a Ficoll–Paque gradient (Amersham, Buckinghamshire, UK) and centrifuged at 2,000 rpm for 30 min without braking. Peripheral blood mononuclear cells (PBMCs), from the interface between the plasma and the Ficoll–Paque gradient, were then collected and washed twice with ice-cold phosphate-buffered saline (PBS), followed by centrifugation at 2,000 rpm for 5 min. Pure MOs were isolated from PBMCs using positive selection with MACS magnetic CD14 antibody (Miltenyi Biotec, Bergisch Gladbach, Germany). Cells were then resuspended in RPMI Medium 1640 (1×) + GlutaMAXTM-1 (Gibco, Thermo Fisher Scientific, Waltham, MA, USA Life Technologies) containing 10 % foetal bovine serum, 100 units/mL penicillin, 100 μg/mL streptomycin and antimycotic. For DC differentiation, the medium was supplemented with 500 U human IL-4 and 800 U GM-CSF (Gentaur Molecular Products, Kampenhout, Belgium). For MAC differentiation, the medium was supplemented with 800 U GM-CSF (Gentaur Molecular Products, Kampenhout, Belgium).

Depending on the amount needed, cells were seeded at a density of 3 × 10^5^ cells/well in 96-well plates, 5 × 10^6^ cells/well in 6-well plates, or 40 × 10^6^ cells in 10-mm plates and cultured for 4 days (unless otherwise noted); medium and cytokines were changed every 2 days. On day 4, cell maturation was induced by culturing cells with 5 μG/ml LPS (Sigma-Aldrich, St. Louis, MI, USA) for 24 h.

The presence of DCs and MACs was checked at the protein level by flow cytometry (Gallios Flow Cytometer, Beckman Coulter) and analysed with FlowJo software (Tree Star, Inc., San Carlos, CA, USA), testing for the upregulation of specific DC markers CD209 conjugated to V450 (BD Horizon, BD Biosciences, Franklin Lakes, NJ, USA), and maturation DC marker CD83 conjugated to APC (Miltenyi Biotec). Expression of CD14 (Miltenyi Biotec), which was high for MOs, medium for MACs and low for DCs, was also confirmed. MACs and DCs were also analysed at the mRNA level with respect to the upregulation of MO marker *CD14* and the following key DC and MAC markers: *CD206*, *CD209*, *CD86*, *CD83*, *MSR1* and *CXCL13*.

### DNA methylation profiling using universal bead arrays

Infinium HumanMethylation450 BeadChips (Illumina, Inc., San Diego, CA, USA) were used to analyse DNA methylation. This array allows >485,000 methylation sites per sample to be interrogated at single-nucleotide resolution. This encompasses 99 % of RefSeq genes, with an average of 17 CpG sites per gene region distributed across the promoter, 5′UTR, the first exon, the gene body and 3′UTR. It covers 96 % of CpG islands, with additional coverage in CpG island shores and the regions flanking them. DNA samples were bisulfite-converted using the EZ DNA methylation kit (Zymo Research, Orange, CA, USA). After bisulfite treatment, the remaining assay steps were performed following the specifications and using the reagents supplied and recommended by the manufacturer. The array was hybridized using a temperature gradient programme, and arrays were imaged using a BeadArray Reader (Illumina Inc.,San Diego, CA, USA). The image processing and intensity data extraction software and procedures were as previously described [9]. Each methylation data point is obtained from a combination of the Cy3 and Cy5 fluorescent intensities from the M (methylated) and U (unmethylated) alleles. Background intensity computed from a set of negative controls was subtracted from each data point. For representation and further analysis we used beta and M values [35]. The beta value is the ratio of the methylated probe intensity to the overall intensity (the sum of the methylated and unmethylated probe intensities). The M value is calculated as the log_2_ ratio of the intensities of the methylated versus unmethylated probe. Beta values range from 0 to 1 and make intuitive sense. They were used to derive heatmaps and for comparisons with DNA methylation percentages from bisulfite pyrosequencing experiments. However, for statistical purposes, the use of M values is more appropriate.

### Detection of differentially methylated CpGs

The approach to selecting differentially methylated CpGs was implemented in the statistical language R. To process Illumina Infinium HumanMethylation450 methylation data we used the methods available in the limma, genefilter and lumi packages, which are accessible from the Bioconductor repository. Before statistical analysis, a pre-processing stage was applied, the main steps being: (1) colour balance adjustment, that is, normalization between two colour channels; (2) quantile normalization based on colour balance-adjusted data; and (3) variance filtering by interquartile range using 0.50 as the threshold value. Results were analysed using an eBayes moderated t-statistical test carried out with the limma package [5]. Specifically, a paired limma was performed as implemented in the IMA package [6]. Several criteria have been proposed to represent significant differences in methylated CpGs. In this study, we considered a probe to be differentially methylated if it had shown a >2-fold (hypermethylation) or <0.5-fold (hypomethylation) difference, and if the statistical test was significant (*p* < 0.01 and FDR < 0.05), using M values for the statistical analysis and cut-off. For candidate gene selection we added the requisite that the difference of beta values was at least 20 %.

### Bisulphite sequencing and pyrosequencing

Bisulphite pyrosequencing was used to validate CpG methylation changes resulting from the analysis with the Infinium HumanMethylation450 BeadChips. Bisulphite modification of genomic DNA isolated from MOs, DCs and MACs was performed using standard methods. Oxidative bisulfite modifications were performed as described recently by Booth and colleagues [36]. The time course was measured in biological triplicates. Briefly, 2 μl of the converted DNA (corresponding to approximately 20–30 ng) were used as a template in each subsequent PCR. Primers for PCR amplification and sequencing were designed with the PyroMark® Assay Design 2.0 software (Qiagen, Hilden, Germany). PCRs were performed with the HotStart Taq DNA polymerase PCR kit (Qiagen, Hilden, Germany), and the success of amplification was assessed by agarose gel electrophoresis. PCR products were pyrosequenced with the PyromarkTM Q24 system (Qiagen, Hilden, Germany). All primer sequences are listed in Additional file 11.

### Gene ontology analysis

GO analysis was performed using the FatiGO tool, which uses Fisher’s exact test to detect significant over-representation of GO terms in one of the sets (list of selected genes) with respect to the other (the rest of the genome). We applied multiple test correction to take into account multiple hypothesis testing (one hypothesis for each GO term), reducing the possibility of false-positive results. GO terms with adjusted values of *p* < 0.05 were considered significant.

### Analysis of TF binding

We used meme tool and the TRANSFAC database to identify STAT6 binding motifs in the 1,000 base pair region upstream and downstream of the centre of hypomethylated CpG sites. ChIP primers were designed for the areas flanking those regions.

### Expression array

Expression studies were performed using the Affymetrix platform according to manufacturer’s instructions. Briefly, 1 μg total RNA was extracted with Trizol from MOs, DCs and MACs, and hybridized to an Affymetrix Human Prime View Array (Affymetrix Inc., Santa Clara, CA, USA). Probe intensity normalization and downstream analysis were obtained using statistical analysis language R in combination with Bioconductor repository functions (http://bioconductor.org). Normalized data obtained with the “affy” package algorithm *vsnrma* [37] was followed by probe identity filtering, under strong statistical confidence thresholds (*p*-value < 0.01; adjusted *p* value (BH) < 0.05; FC < 2 & FC > 2 for downregulated and upregulated respectively). Finally, comparison of expression and DNA methylation data were performed by applying custom R scripting.

### Graphs and heatmaps

All graphs were created using Prism5 Graphpad. Heatmaps of the expression or methylation data were generated using the Genesis program (Graz University of Technology, Graz, Austria).

### BrdU proliferation assays

BrdU was used at a final concentration of 300 μM, as previously described [9]. BrdU pulsing solution was added to each well at days 2 and 4. For flow cytometry assays, CD14+ cells were seeded in 6-well plates and cultured in differentiation media. BrdU was added to the medium at different times and after 2 days cells were fixed (4 % paraformaldehyde, 30 minutes, room temperature), permeabilized (PBS-bovine serum albumin-Triton X-100 0.8 %, 10 min, room temperature), and treated with HCl 2 N for 30 min. After DNA opening, HCl was neutralized by two 5-min washes with NaBo (0.1 M, pH 8.5) and two 5-min washes with PBT (PBS-bovine serum albumin-Triton X-100 0.8 %). Cells were incubated with anti-BrdU antibody (18 h at 4 °C, 1:1,000 dilution) and an anti-mouse Alexa-488 conjugated antibody was added to detect the BrdU-positive nuclei.

### Transfection of primary human MOs

We used ON-TARGETplus siRNAs against STAT6, JAK3 and TET2 to perform knockdown experiments in peripheral blood MOs. We also used ON-TARGETplus Non-targeting Control Pool as a negative control. For PU.1 silencing experiments, two different Silencer® select pre-designed siRNAs against human PU.1 (one targeting exon 2 and another targeting the 3′UTR) and a Silencer® select negative control were used. We transfected MOs with siRNAs using Lipofectamine 3000 Reagent (Thermo Fisher Scientific Co., Carlsbad, CA, USA) and added cytokines 24 h later. We refreshed the transfection 3 days after starting the culture. We examined the levels of the target proteins by western blot 2 and 4 days after siRNA transfection. Three biological replicates of the experiments were performed.

### Chromatin immunoprecipitation assays

For ChIP assays, CD14+ cells (MOs) treated with IL-4/GM-CSF for 0 and 2 days were crosslinked with 1 % formaldehyde and subjected to immunoprecipitation after sonication. ChIP experiments were performed using a low cell ChIP kit (Diagenode SA, Seraing, Belgium). They were analysed by real-time quantitative PCR. Data are represented as the ratio of the bound fraction to the input for each specific factor. We used an antibody against STAT6 (Santa Cruz, sc-981x), and histone marks H3K9me3 (Abcam, ab8898) and H3K27me3 (Millipore 07-449). Human IgG was used as a negative control. Primer sequences were designed to contain predicted or known TF binding (from TRANSFAC or ChIPseq data) as close as possible to the CpG undergoing methylation changes. Primer sequences are shown in Additional file 11. Three biological replicates of the experiments were performed.

### Inhibition of the JAK3

JAK3 was pharmacologically inhibited using the specific inhibitor PF-956980 [24] (Sigma) following the manufacturer’s instructions. MOs were pre-treated for 1 h with PF-956980 on day 0 of differentiation. Following pre-incubation, MO differentiation was induced with IL-4/GM-CSF or GM-CSF alone in the presence of PF-956980.

### STAT6 constructs, generation of lentiviral supernatants and cellular transduction

We amplified the STAT6 coding DNA sequence using PCR with AccuPrime Pfx high-fidelity DNA Polymerase (Invitrogen, Thermo Fisher Scientific, Waltham, MA, USA) following the manufacturer’s instructions. A reverse primer containing an HA tag was introduced in the sequence N-terminal end. The double mutant STAT6VT was prepared by point mutagenesis using PCR to introduce two alanine residues at amino acid positions 547/548. Sequences were subcloned in pCDH-MIG vector and verified by sequencing.

Two culture supernatants were generated by transient transfection of 293FT cells and were collected 48 and 72 h post-transfection. The first supernatant contained the pMSCV-GFP (mock) or the pMSCV-GFP-STAT6VT, and the second supernatant contained the SIVmac-derived helper particles that pack the Vpx protein able to degrade SAMHD1 [38]. Viral supernatants were concentrated × 10 by ultracentrifugation at 20,000 rpm at 4 °C for 2 h using Sorvall centrifuge (Thermo Scientific) and fresh MOs were infected with both viral supernatants. Infected MOs were cultured with GM-CSF for 9 days at 37 °C. The media was refreshed every 2 days. GFP-positive cells were sorted in a MoFlo Astrios (Beckman Coulter, Brea, CA, USA), lysed in Proteinase K buffer and incubated overnight (ON) at 65 °C. Genomic DNA was isolated by standard phenol-chloroform extraction for bisulphite pyrosequencing.

### Data Access

Methylation array and expression array data for this publication have been deposited in the NCBI Gene Expression Omnibus and are accessible through GEO Series accession number: [GEO: GSE71837] (methylation data for DC and MAC differentiation, corresponding to Fig. 1c), [GEO: GSE75937] (methylation data for DC and MAC differentiation in the presence of JAK3 inhibitors, corresponding to Fig. 3e) and [GEO: GSE75938] (expression data corresponding to Fig. 2a).

## Additional files

Additional file 1:
**(A) DC and MAC markers, checked by quantitative RT-PCR.** Upregulation of DC and MAC markers *CD209*, *CD83*, *MSR1*, *CXCL13*, *CD206*, *CD86* and downregulation of monocyte marker *CD14* were detected. (B) Specific DC and MAC surface markers analysed by flow cytometry. The CD14 receptor is high in MOs, intermediate in MACs and low/negative in DCs. CD209 is a DC marker; CD206 is positive in DCs and MACs; CD83 is increased in mDCs; CD86 is increased in mMACs. The percentage of positive cells for each marker is indicated in each graph. Because CD83 and CD86 are also present in the immature cells, we have established ‘highly positive cells’ once a significant shift is observed, and a second percentage is indicated for those highly positive cells (bottom). (C) BrdU assay showing absence of proliferation during dendritic and macrophage differentiation. (PDF 113 kb) Additional file 2:
**List of hypomethylated and hypermethylated genes during MO to DC and MAC differentiation and maturation (>2-fold or <0.5-fold change;** ***p*** **< 0.01 and FDR < 0.05).** Difference of β values are indicated for each gene. (XLSX 1035 kb) Additional file 3:
**(A) Scatterplots showing DNA methylation profiles of matching pairs (MO-iDC; MO-iMAC; iDC-mDC; iMAC-mMAC).** CpGs with significant differences (>2-fold change or <0.5-fold change; *p* < 0.01 and FDR < 0.05) in average results for three samples are highlighted in blue (DCs) or red (MACs). The x-axis and y-axis of each graph correspond to the β values for each paired comparison, as indicated in the lower left corner. (B) A Venn diagram showing the overlap between Zhang et al. [17] data and our own data corresponding to the list of demethylated genes in MO-to-iDC differentiation. (C) Technical validation of the array data by bisulfite pyrosequencing of modified DNA. Three groups of genes are represented: demethylated genes specific to iDC differentiation, demethylated genes specific to iMAC differentiation, and genes that are commonly demethylated in iDC and iMAC differentiation. (D) 5hmC content in several of the CpGs that are rapidly demethylated after cytokine addition to MOs. (PDF 1037 kb) Additional file 4:
**Selected DC-specific (blue), MAC-specific (red) and common to DC and MAC (grey) demethylated genes during DC and MAC differentiation.** (DOCX 22 kb) Additional file 5:
**Role of TET2 in DNA demethylation and acquisition of DC phenotype.** (A) Flow cytometry analysis of CD14, CD209 and CD83 in DCs and MACs transfected with an siRNA against TET2, and their corresponding siRNA negative control. (B) Time-course analysis of the effects of TET2 silencing on DNA methylation changes during both DC (right panel) and MAC (left panel) differentiation. Specific DC (upper panel), MAC (bottom panel) and common (bottom panel) genes for both DC and MAC differentiation were analysed. (PDF 23 kb) Additional file 6:
**Relationship between methylation and expression changes in MAC and DC differentiation.** “Poised” genes are those undergoing DNA methylation changes at the differentiation stage and expression changes only at the activation stage. (XLSX 640 kb) Additional file 7:
**(A) DNA methylation dynamics of selected loci during MO-to-DC and MO-to-MAC differentiation and maturation.** Methylation percentage determined by bisulfite pyrosequencing. (B) RNA expression dynamics of selected loci during MO-to-DC and MO-to-MAC differentiation and maturation. Quantitative RT-PCR data relative to HPRT1 and RPL38. (C) ChIP assays of IL1A (MAC-specific) and AIM2 (common; displaying higher LPS-mediated upregulation in MACs) with anti-histone H3K27me3 and anti-histone H3K9me3 in MOs, and in a time-course manner in differentiation to iDCs and iMACs, as well as mDCs and mMACs (120 h + LPS). (PDF 54 kb) Additional file 8:
**(A) Surface DC and MAC markers analysed by flow cytometry during DC and MAC differentiation in the presence of the inhibitor against JAK3 or the carrier (DMSO).** (B) Effects of JAK3 inhibition on gene expression of specific DC genes regulated by DNA methylation in DC and MAC population. Quantitative reverse transcriptase polymerase chain reaction (RT-PCR) data relative to HPRT1 and RPL38. (C) Effects of JAK3 inhibition by PF-956980 on DNA methylation over time in MAC (GM-CSF) differentiation, focusing on two DC-specific genes (top), MAC-specific genes (middle) and two genes demethylated in both DC and MAC differentiation (bottom). (D) Cluster analysis showing the effects on distance between samples following treatment with JAK3 inhibitor PF-956980 on MOs exposed for 96 h to GM-CSF/IL-4 or GM-CSF alone. (E) Beta values extracted from the high-throughput analysis of selected genes in MOs, iDCs, and iMACs in the absence or presence of PF-956980. (PDF 57 kb) Additional file 9:
**(A) Flow cytometry was used to detect surface marker changes in siRNA experiments during DC (-si STAT6 and -si JAK3) and MAC (-si STAT6) differentiation and maturation.** A negative control pool from Dharmacon was used as a control in all experiments performed. (B) DNA methylation of MO transfected cells with an siRNA against STAT6 and their negative control, in the absence of IL-4 during MO differentiation. (C) Gene expression consequences measured by quantitative RT-PCR in DC, when STAT6 is inhibited by an siRNA. (PDF 26 kb) Additional file 10:
**PU.1. contributes to DNA demethylation.** (A) Western blot showing decrease in PU.1 levels by treating cells with an –si against PU.1. (B) Analysis of DC phenotype (CD14, CD209 and CD83 expression) during MO differentiation in PU.1-silenced cells. (C) Time-course analysis of DNA methylation in cells treated with -si against PU.1. DCs (right panel) and MACs (left panel) were analysed for specific DC (upper panel), MAC and common (bottom panels) genes during differentiation of MOs into both DCs and MACs. (PDF 26 kb) Additional file 11:
**List of primers.** (XLSX 14 kb)

## Abbreviations

ChIP: chromatin immunoprecipitation
DC: dendritic cell
FDR: false discovery rate
GFP: green fluorescent protein
GM-CSF: granulocyte-macrophage colony-stimulating factor
GO: gene ontology
iDC: immature dendritic cell
IL-4: interleukin 4
IL-4R: interleukin 4 receptor
iMAC: immature macrophage
JAK3: Janus kinase 3
LPS: lipopolysaccharide
MAC: macrophage
mDC: mature dendritic cell
mMAC: mature macrophage
MO: monocyte
OC: osteoclast
PBS: phosphate-buffered saline
TF: transcription factor
TLR: Toll-like receptor
TSS: transcription start site
