# Supplementary figures and images for: IL-4 orchestrates STAT6-mediated DNA demethylation leading to dendritic cell differentiation
Source: Genome Biol. 2016 Jan 13;17:4. doi: 10.1186/s13059-015-0863-2 (PMC4711003; doi:10.1186/s13059-015-0863-2)

A

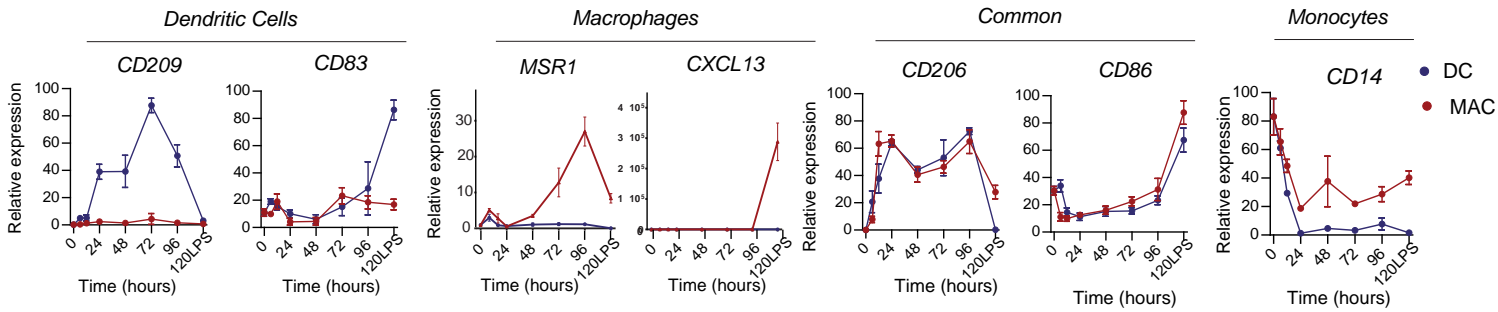

B

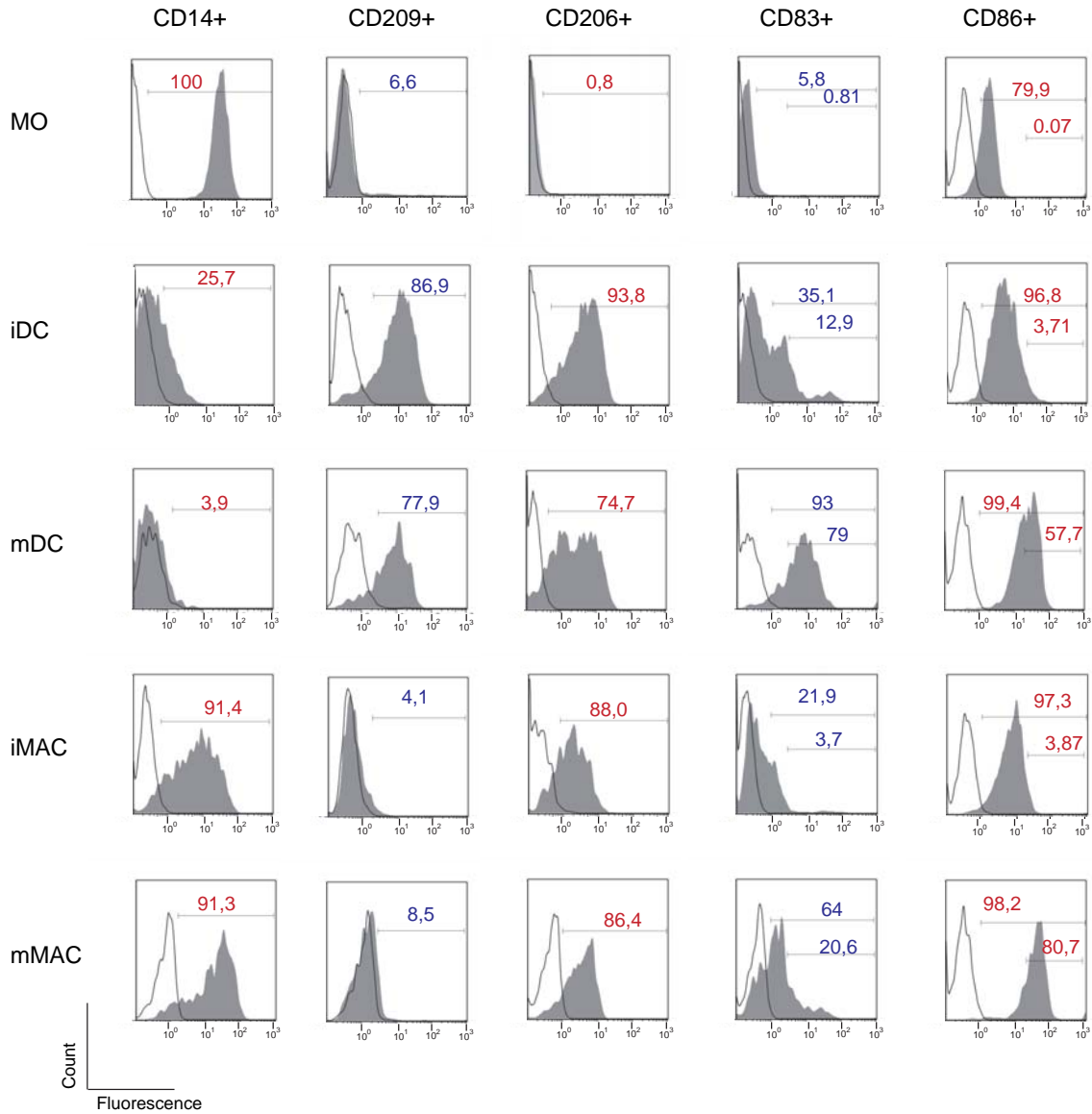

C

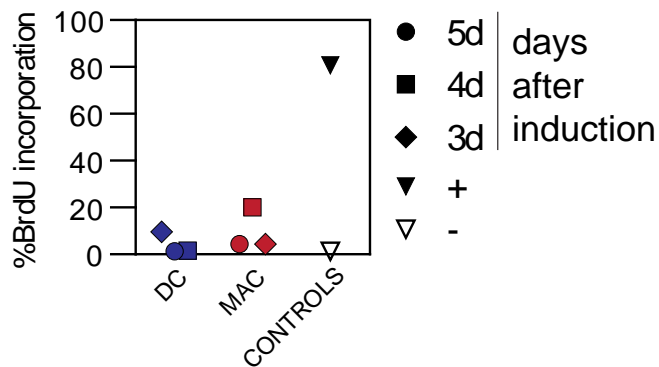

Supplement: Additional file 1: — (A) DC and MAC markers, checked by quantitative RT-PCR. Upregulation of DC and MAC markers CD209, CD83, MSR1, CXCL13, CD206, CD86 and downregulation of monocyte marker CD14 were detected. (B) Specific DC and MAC surface markers analysed by flow cytometry. The CD14 receptor is high in MOs, intermediate in MACs and low/negative in DCs. CD209 is a DC marker; CD206 is positive in DCs and MACs; CD83 is increased in mDCs; CD86 is increased in mMACs. The percentage of positive cells for each marker is indicated in each graph. Because CD83 and CD86 are also present in the immature cells, we have established ‘highly positive cells’ once a significant shift is observed, and a second percentage is indicated for those highly positive cells (bottom). (C) BrdU assay showing absence of proliferation during dendritic and macrophage differentiation. (PDF 113 kb) [file 13059_2015_863_MOESM1_ESM.pdf]

A

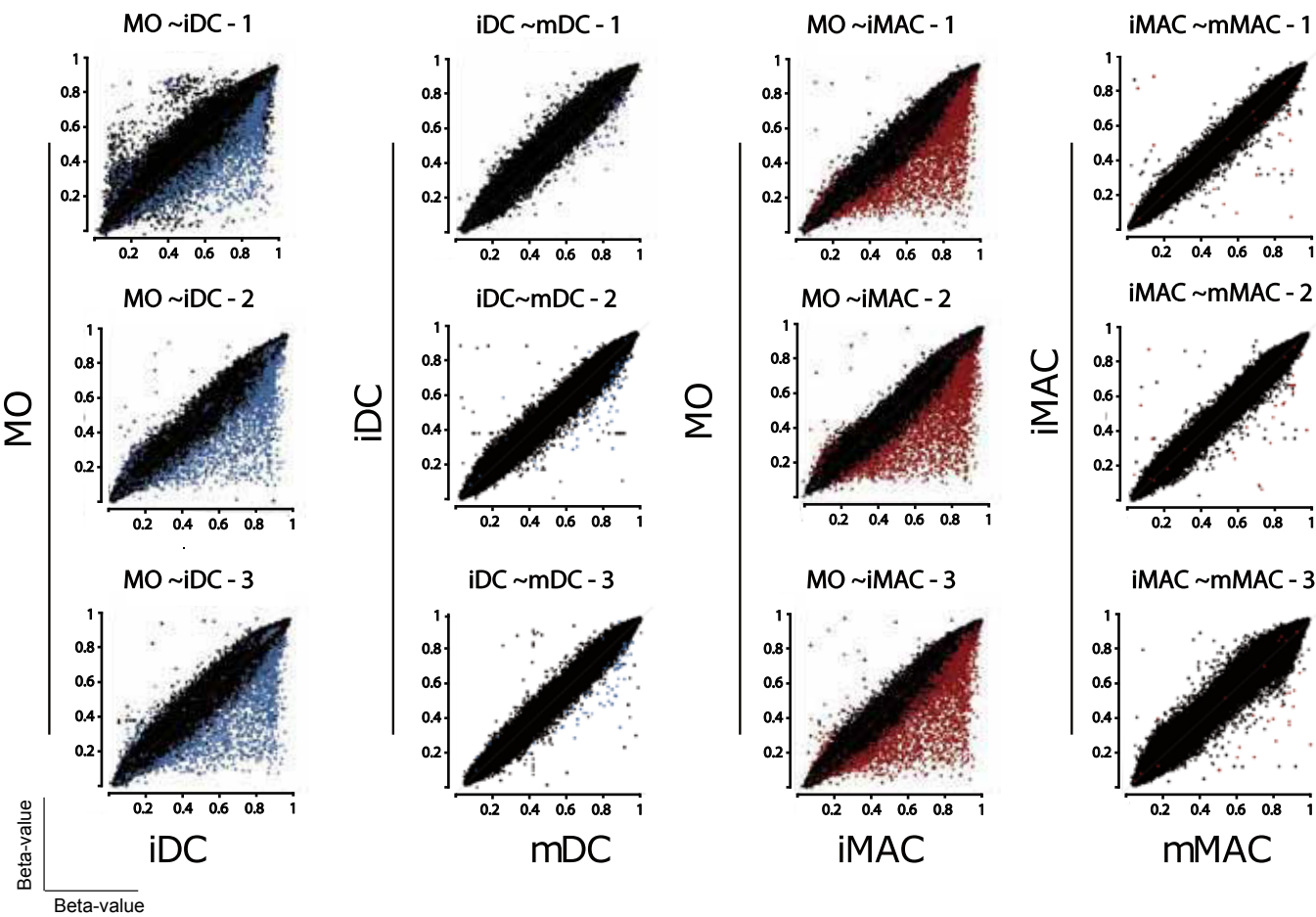

B

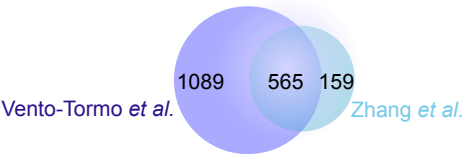

D

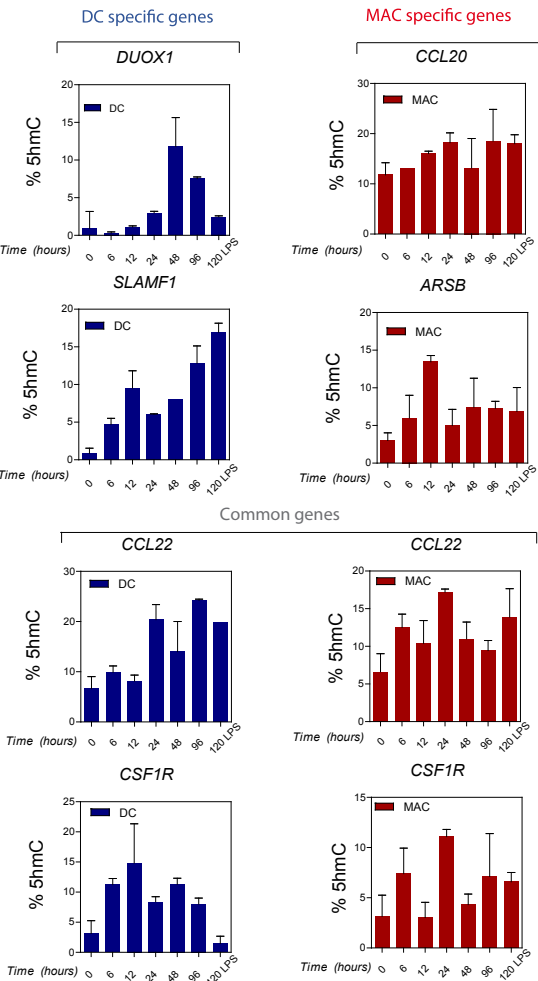

C

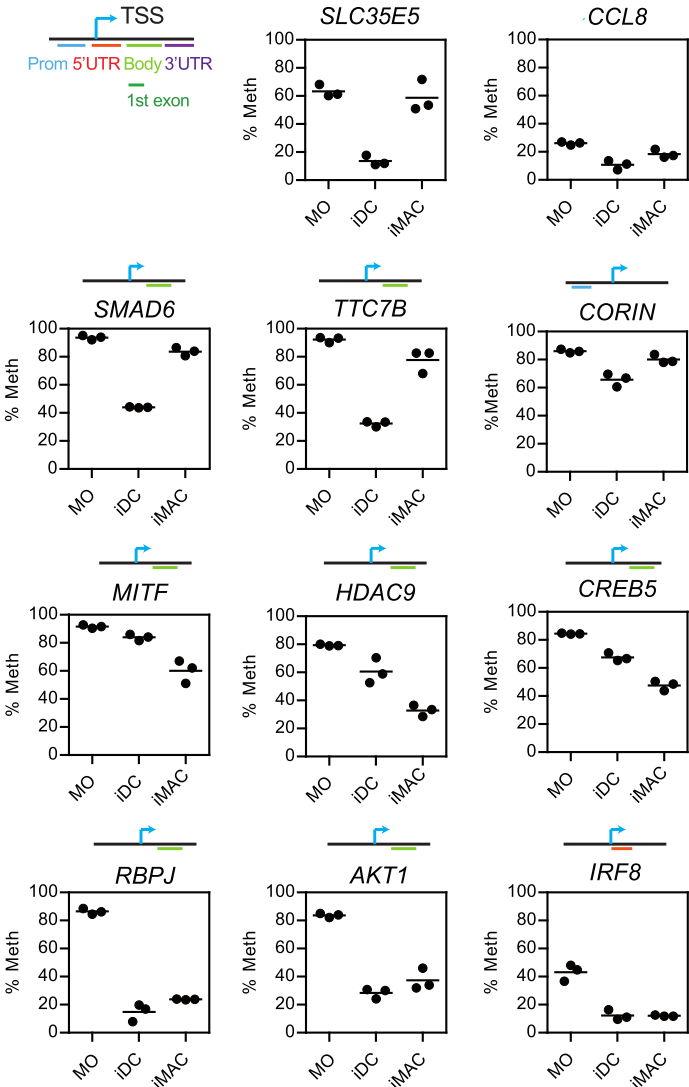

Supplement: Additional file 3: — (A) Scatterplots showing DNA methylation profiles of matching pairs (MO-iDC; MO-iMAC; iDC-mDC; iMAC-mMAC). CpGs with significant differences (>2-fold change or <0.5-fold change; p < 0.01 and FDR < 0.05) in average results for three samples are highlighted in blue (DCs) or red (MACs). The x-axis and y-axis of each graph correspond to the β values for each paired comparison, as indicated in the lower left corner. (B) A Venn diagram showing the overlap between Zhang et al. [17] data and our own data corresponding to the list of demethylated genes in MO-to-iDC differentiation. (C) Technical validation of the array data by bisulfite pyrosequencing of modified DNA. Three groups of genes are represented: demethylated genes specific to iDC differentiation, demethylated genes specific to iMAC differentiation, and genes that are commonly demethylated in iDC and iMAC differentiation. (D) 5hmC content in several of the CpGs that are rapidly demethylated after cytokine addition to MOs. (PDF 1037 kb) [file 13059_2015_863_MOESM3_ESM.pdf]

A

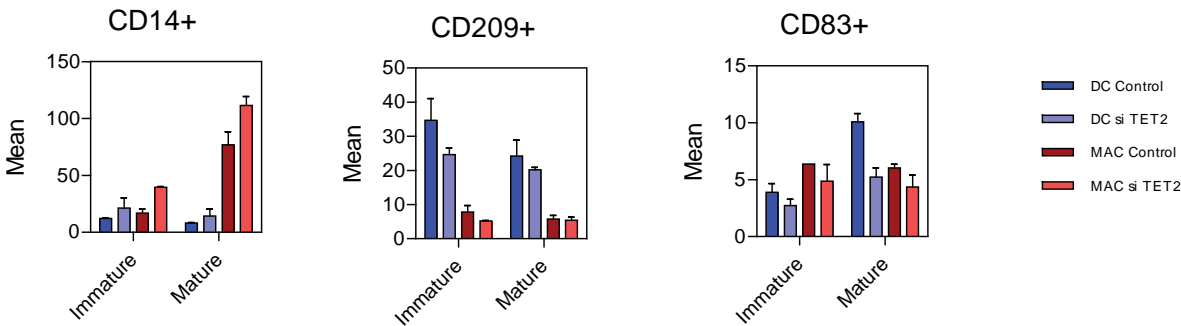

B

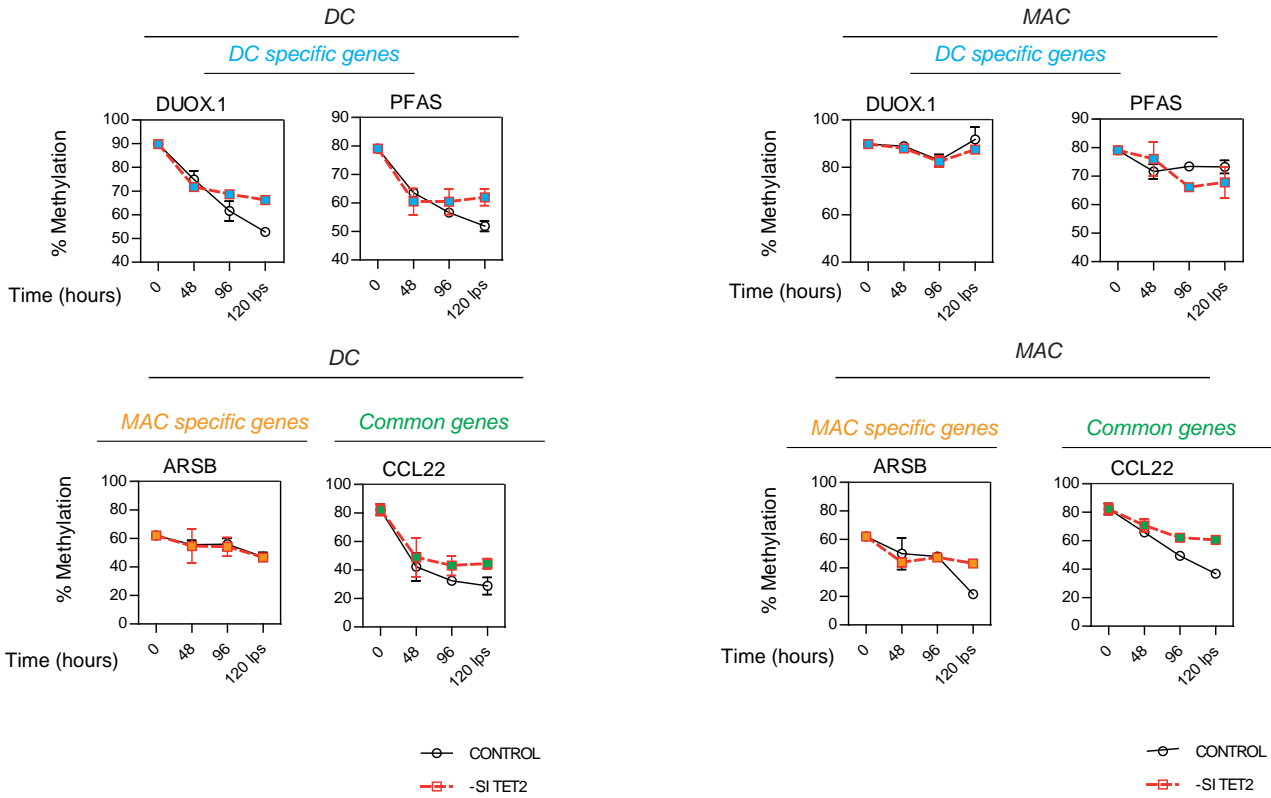

Supplement: Additional file 5: — Role of TET2 in DNA demethylation and acquisition of DC phenotype. (A) Flow cytometry analysis of CD14, CD209 and CD83 in DCs and MACs transfected with an siRNA against TET2, and their corresponding siRNA negative control. (B) Time-course analysis of the effects of TET2 silencing on DNA methylation changes during both DC (right panel) and MAC (left panel) differentiation. Specific DC (upper panel), MAC (bottom panel) and common (bottom panel) genes for both DC and MAC differentiation were analysed. (PDF 23 kb) [file 13059_2015_863_MOESM5_ESM.pdf]

**A** **B**

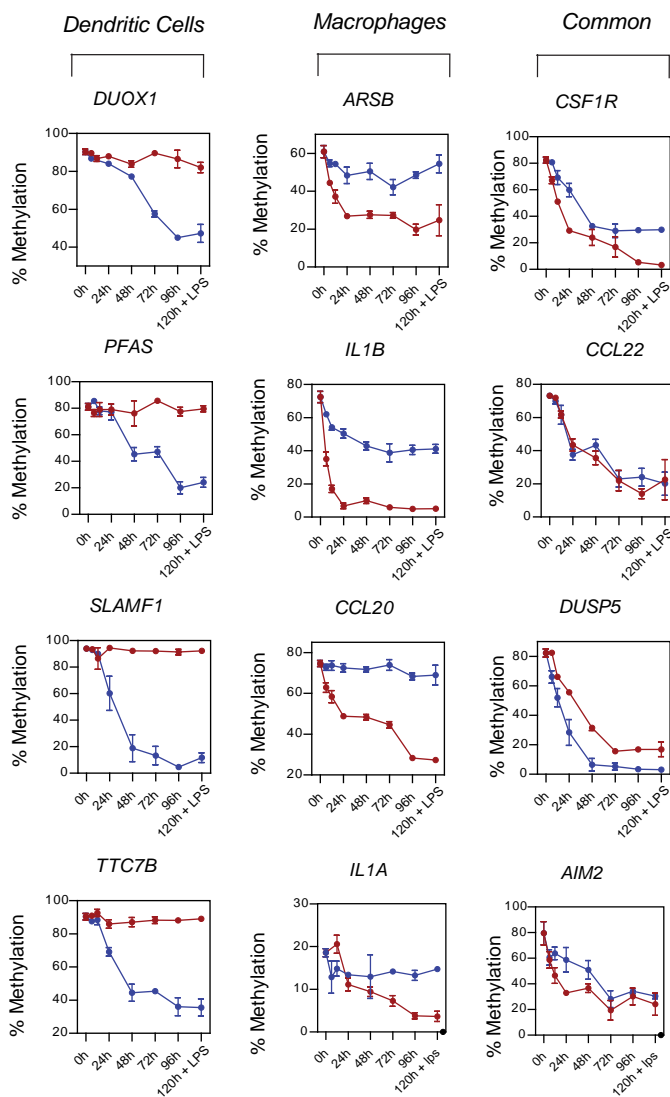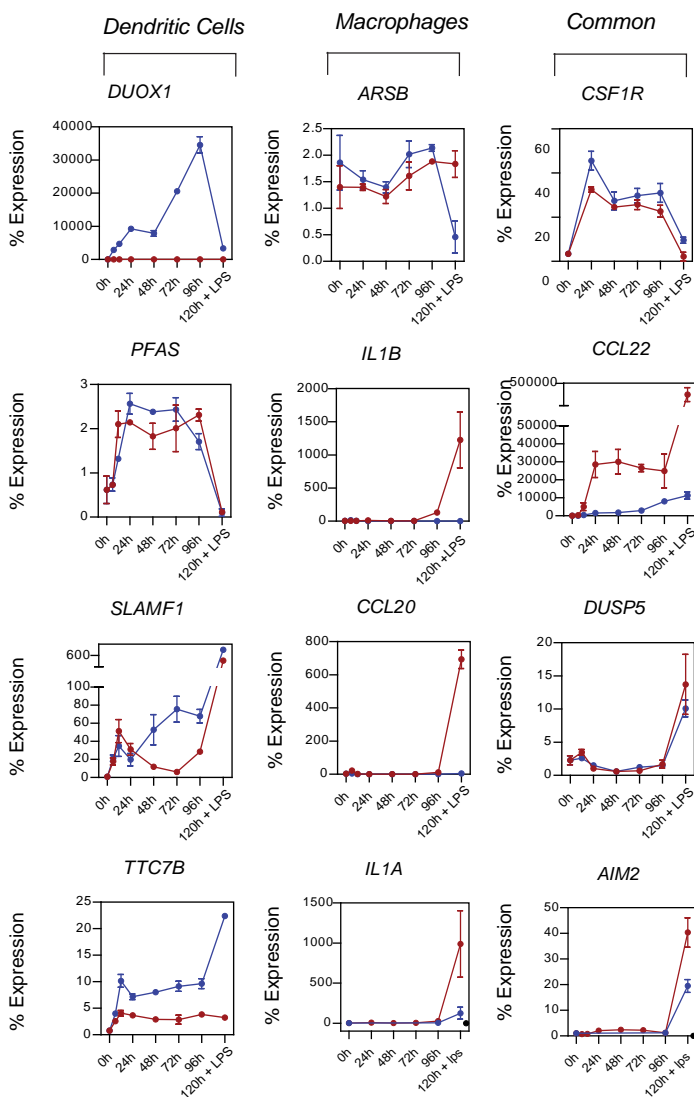

**C**

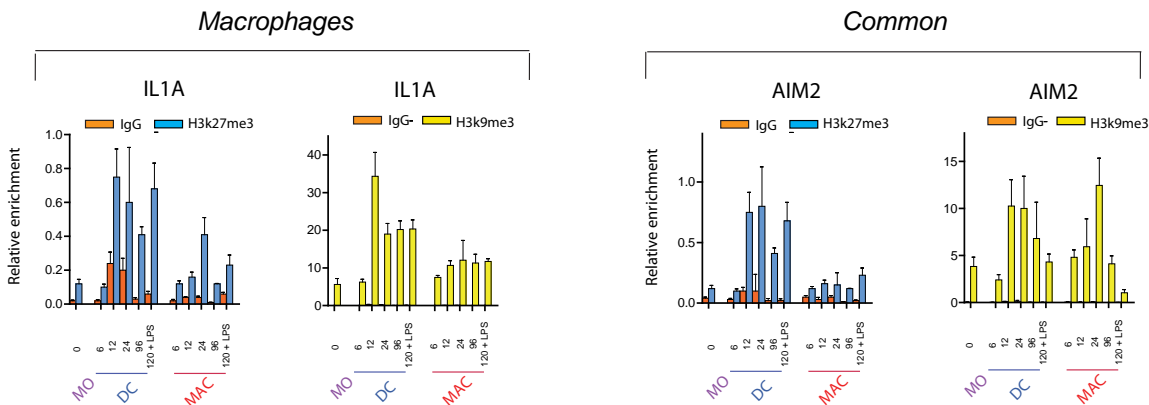

Supplement: Additional file 7: — (A) DNA methylation dynamics of selected loci during MO-to-DC and MO-to-MAC differentiation and maturation. Methylation percentage determined by bisulfite pyrosequencing. (B) RNA expression dynamics of selected loci during MO-to-DC and MO-to-MAC differentiation and maturation. Quantitative RT-PCR data relative to HPRT1 and RPL38. (C) ChIP assays of IL1A (MAC-specific) and AIM2 (common; displaying higher LPS-mediated upregulation in MACs) with anti-histone H3K27me3 and anti-histone H3K9me3 in MOs, and in a time-course manner in differentiation to iDCs and iMACs, as well as mDCs and mMACs (120 h + LPS). (PDF 54 kb) [file 13059_2015_863_MOESM7_ESM.pdf]

A

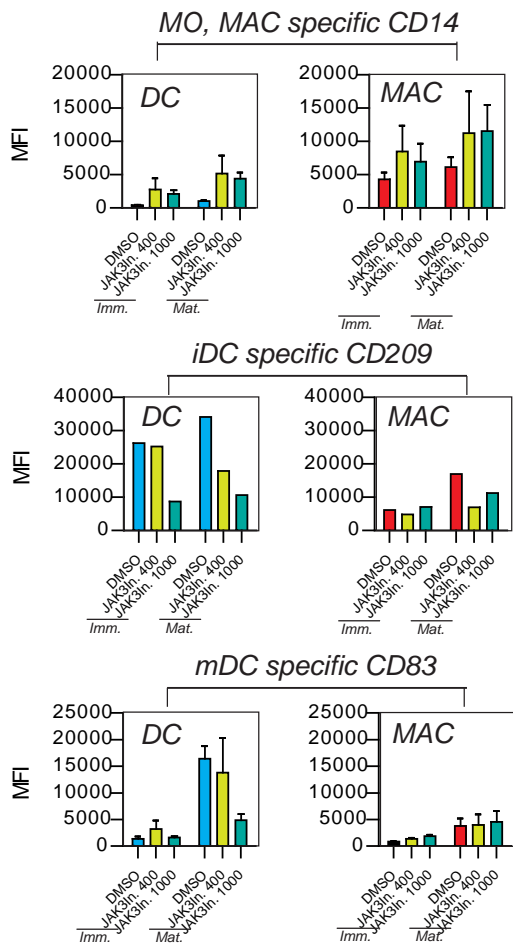

B

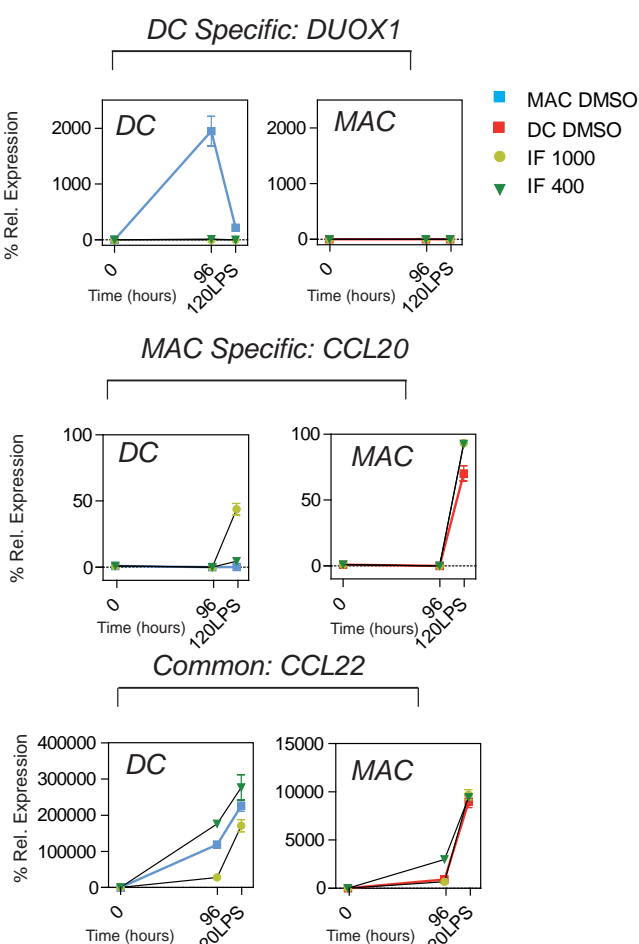

C

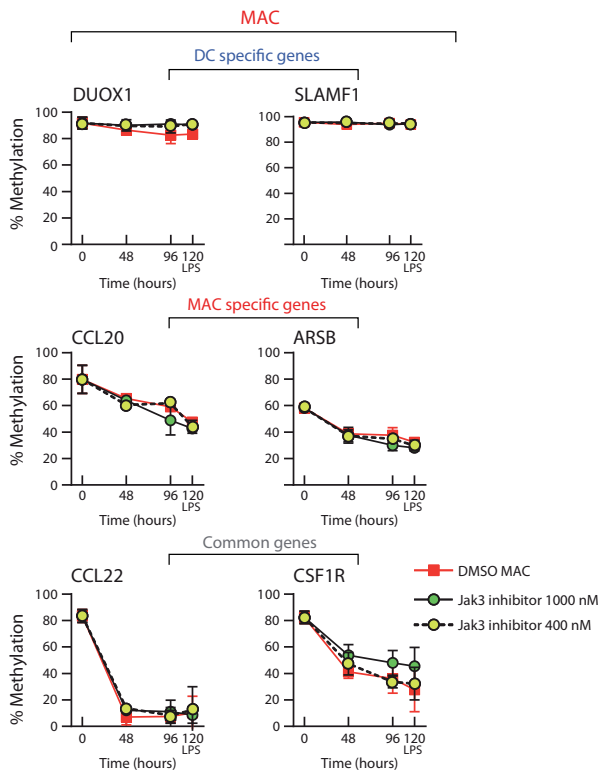

D

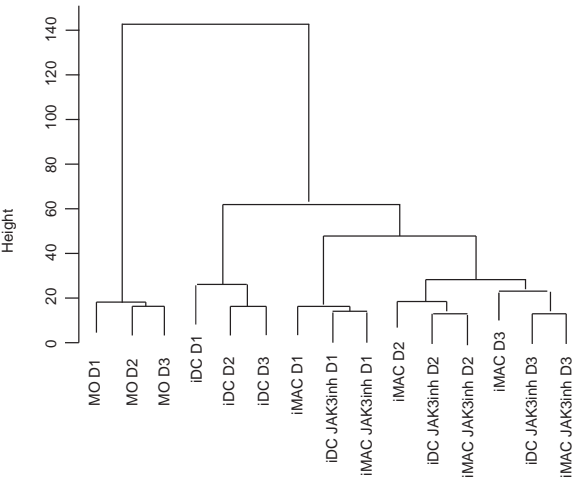

E

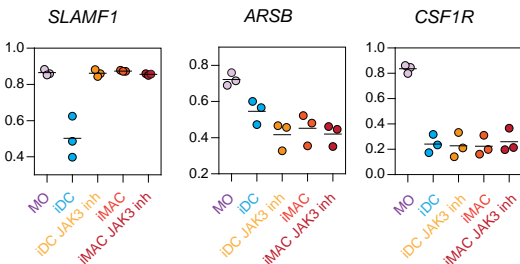

Supplement: Additional file 8: — (A) Surface DC and MAC markers analysed by flow cytometry during DC and MAC differentiation in the presence of the inhibitor against JAK3 or the carrier (DMSO). (B) Effects of JAK3 inhibition on gene expression of specific DC genes regulated by DNA methylation in DC and MAC population. Quantitative reverse transcriptase polymerase chain reaction (RT-PCR) data relative to HPRT1 and RPL38. (C) Effects of JAK3 inhibition by PF-956980 on DNA methylation over time in MAC (GM-CSF) differentiation, focusing on two DC-specific genes (top), MAC-specific genes (middle) and two genes demethylated in both DC and MAC differentiation (bottom). (D) Cluster analysis showing the effects on distance between samples following treatment with JAK3 inhibitor PF-956980 on MOs exposed for 96 h to GM-CSF/IL-4 or GM-CSF alone. (E) Beta values extracted from the high-throughput analysis of selected genes in MOs, iDCs, and iMACs in the absence or presence of PF-956980. (PDF 57 kb) [file 13059_2015_863_MOESM8_ESM.pdf]

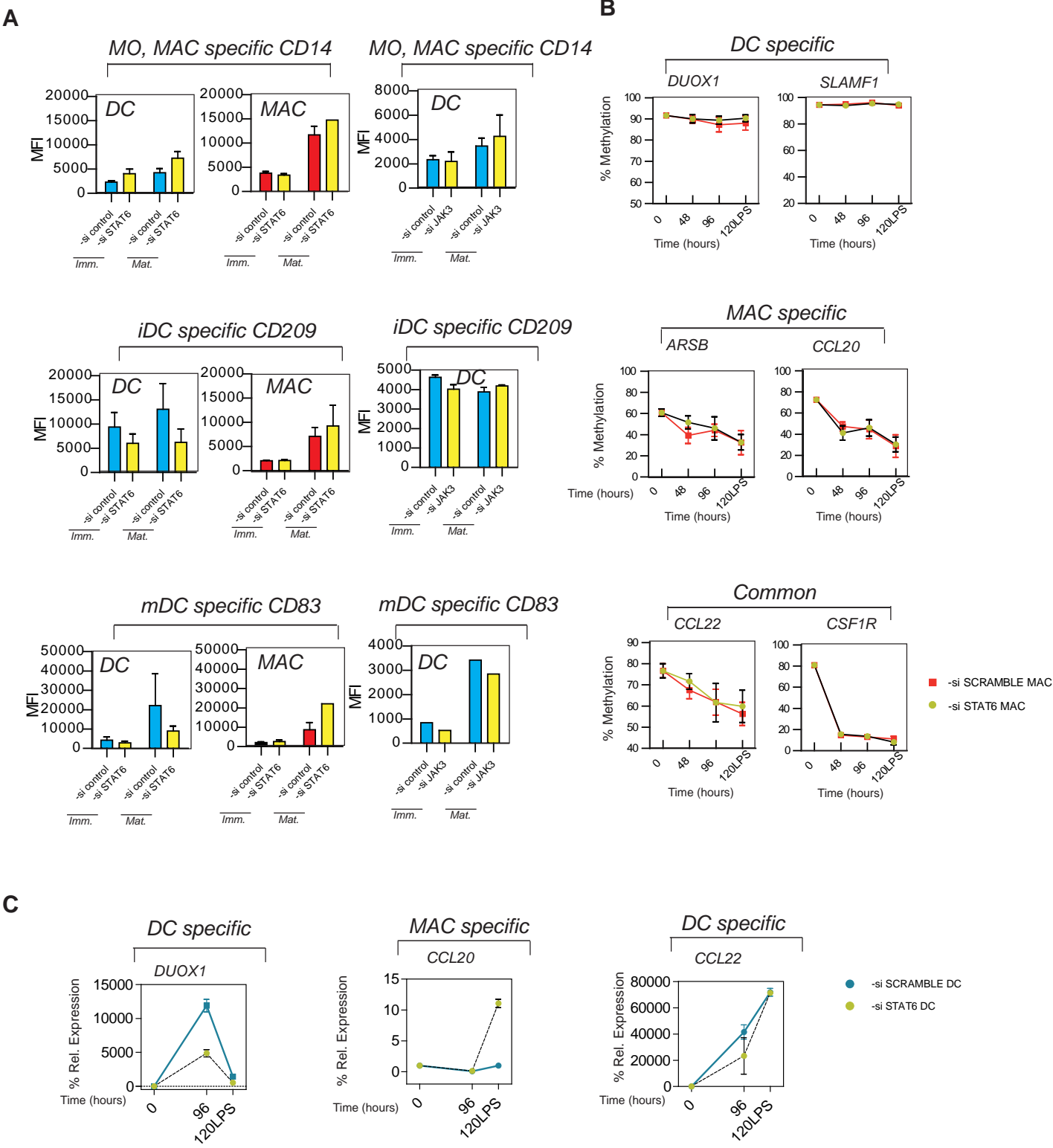

Supplement: Additional file 9: — (A) Flow cytometry was used to detect surface marker changes in siRNA experiments during DC (-si STAT6 and -si JAK3) and MAC (-si STAT6) differentiation and maturation. A negative control pool from Dharmacon was used as a control in all experiments performed. (B) DNA methylation of MO transfected cells with an siRNA against STAT6 and their negative control, in the absence of IL-4 during MO differentiation. (C) Gene expression consequences measured by quantitative RT-PCR in DC, when STAT6 is inhibited by an siRNA. (PDF 26 kb) [file 13059_2015_863_MOESM9_ESM.pdf]

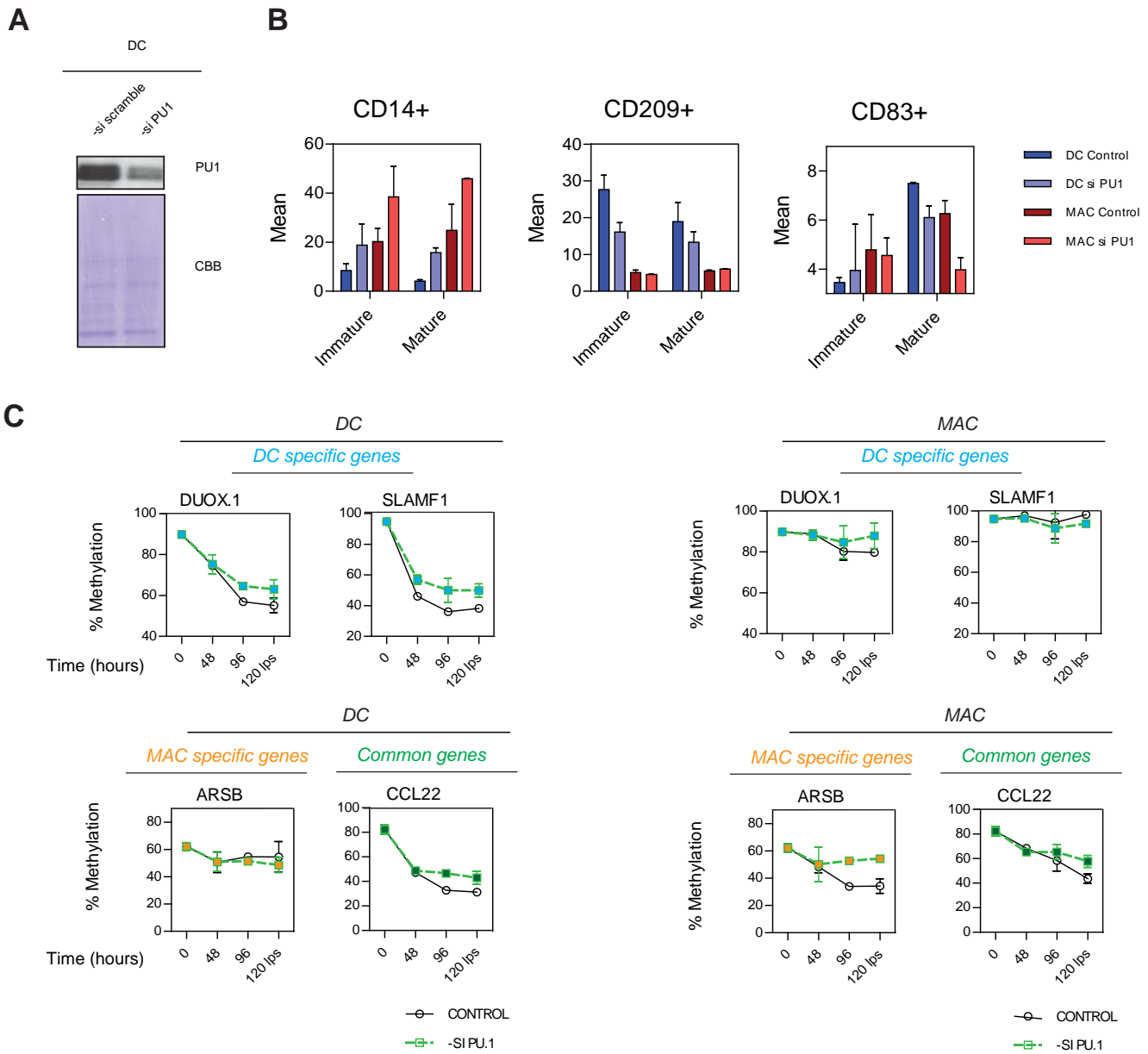

Supplement: Additional file 10: — PU.1. contributes to DNA demethylation. (A) Western blot showing decrease in PU.1 levels by treating cells with an –si against PU.1. (B) Analysis of DC phenotype (CD14, CD209 and CD83 expression) during MO differentiation in PU.1-silenced cells. (C) Time-course analysis of DNA methylation in cells treated with -si against PU.1. DCs (right panel) and MACs (left panel) were analysed for specific DC (upper panel), MAC and common (bottom panels) genes during differentiation of MOs into both DCs and MACs. (PDF 26 kb) [file 13059_2015_863_MOESM10_ESM.pdf]
