# Supplementary material for: IL-4 orchestrates STAT6-mediated DNA demethylation leading to dendritic cell differentiation
Source: Genome Biol. 2016 Jan 13;17:4. doi: 10.1186/s13059-015-0863-2 (PMC4711003; doi:10.1186/s13059-015-0863-2)
Supplement: Additional file 4: — Selected DC-specific (blue), MAC-specific (red) and common to DC and MAC (grey) demethylated genes during DC and MAC differentiation. (DOCX 22 kb) [file 13059_2015_863_MOESM4_ESM.docx]

| Additional File 4. Selected DC- (blue) and MAC- (red) specific and common to DC and MAC (grey) demethylated genes durig DC and MAC differentiation | | | | | | | |  |  |
| --- | --- | --- | --- | --- | --- | --- | --- | --- | --- |
| Gene | CpG number | | Localization | | LogFC [DC,MAC] | Diff. beta  [DC,MAC] | Relevance of gene products in inflammatory context |  |  |
| *DUOX1* | | cg13570892 | | Body | [-2.62;-] | [-0.28;-] | Protein from NADPH family, involved in lactoperoxidase-mediated defense | |  |
| *SLAMF1* | | cg26883963 | | 3'UTR | [-4.03;-] | [-0.58;-] | Receptor involved in T and B cell activation, and promotes DC maturation | |  |
| *PFAS* | | cg00194780 | | 3'UTR | [-2.25;-] | [-0.33;-] | Enzyme involved in purine biosynthesis | |  |
| *SMAD6*  *CORIN*  *CCL8*  *TAL1*  *RUNX1* | | cg01339004  cg02955940  cg01636591  cg00408773  cg02869559  cg12477880 | | Body  TSS1500  1stExon;5'UTR  Body  Body  1stExon;Body | [-2.42;-]  [-1.98,-]  [-1.33,-]  [-1.02,-]  [-1.00;-]  [-1.02;-] | [-0.29;-]  [-0.25;-]  [-0.20;-]  [-0.15;-]  [-0.15;-]  [-0.15;-] | Mediator of TGF beta antinflammatory activity  Serine protease class of the trypsin superfamily.  Chemotactic factor, pro-1nflammatory  Transcription factor involved in hematopoyesis.  Transcription factor involved in hematopoyesis. | |  |
| *ARSB* | | cg07379703 | | TSS1500 | [-;-2.13] | [-0.35;-] | Hydrolisis of sulfate groups of N-Acetyl-D-galactosamine, chondriotin sulfate, and dermatan sulfate | |  |
|  | |  | |  |  |  |  | |  |
| *CCL20* | | cg08575688 | | TSS200 | [-;-1.09] | [-0.17;-] | Chemotactic factor | |  |
|  | |  | |  |  |  |  | |  |
| *IL1B* | | cg14117934 | | 3'UTR | [-;-2.54] | [-0.39;-] | Proinflammatory cytokine and pyrogen | |  |
| *MITF* | | cg04811592 | | Body | [-;-2.78] | [-0.31;-] | Transcription factor involved in myeloid differentiation | |  |
| *NLRC5*  *HDAC9*  *CASP1* | | cg08159663  cg04892643  cg17008031 | | TSS1500  Body  TSS1500 | [-;-1.27]  [-;-2.38]  [-;-1.44] | [-0.21;-]  [-0.35;-]  [-0.19;-] | Inflammasome sensing protein  Histone deacetylase  Gene involved in activate the inactive precursor of interleukin-1. | |  |
| *CSF1R* | | cg07260017  cg12974258 | | 5'UTR  TSS200 | [-3.81;-3.85]  [-1.29;-2.18] | [-0.57;-0.58]  [-0.19;-0,19] | M-CSF receptor | |  |
| *CCL22* | | cg23471482  cg04250732 | | TSS1500  TSS1500 | [-3.22;-3.29]  [-1.31;-1.51] | [-0.50;-0.50]  [-0.20;-0.20] | Chemotactic factor | |  |
| *DUSP5* | | cg10080966 | | Body | [-3.75;-2.82] | [-0.56;-0.57] | Phosphatase that negatively regulates members of the mitogen-activated protein (MAP) kinase superfamily (MAPK/ERK,SAPK/JNK,p38) | |  |
| *IRF8* | | cg07955474 | | 5'UTR | [-2.09;-2.41] | [-0.33;-0.33] | Transcription factor that regulates myeloid differentiation | |  |
| *RBPJ* | | cg04722169 | | Body | [-3.03;-2.29] | [-0.38;-0.38] | Transcriptional regulator from the Notch pathway | |  |
| *AKT1* | | cg26099837  cg15912732  cg12789068  cg04971812 | | Body  Body  Body  3'UTR | [-5.82;-5.72]  [-5.33;-4.70]  [-1.88;-2.03]  [-1.16;-1.42] | [-0.73;-0.73]  [-0.71;-0.71]  [-0.24;-0.24]  [-0.09;-0.09] | Serine/threonine kinase that regulate many processes including metabolism, proliferation, cell survival, growth and angiogenesis | |  |
| *CREB5*  *IL1RN* | | cg09930712  cg11783497 | | Body  TSS200 | [-1.17;-2.65]  [-2.63;-3.18] | [-0.10;-0.10]  [-0.38;-0.38] | Member of the CRE (cAMP response element)-binding protein family.  Member of the interleukin 1 cytokine family. Inhibits the activities of interleukin 1, alpha and beta, and modulated a variety of interleukin 1 related immune and inflammatory responses. | |  |
|  | |  | |  |  |  |  | |  |
|  | |  | |  |  |  |  | |  |
